# Nrf2 suppresses macrophage inflammatory response by blocking proinflammatory cytokine transcription

**DOI:** 10.1038/ncomms11624

**Published:** 2016-05-23

**Authors:** Eri H. Kobayashi, Takafumi Suzuki, Ryo Funayama, Takeshi Nagashima, Makiko Hayashi, Hiroki Sekine, Nobuyuki Tanaka, Takashi Moriguchi, Hozumi Motohashi, Keiko Nakayama, Masayuki Yamamoto

**Affiliations:** 1 Department of Medical Biochemistry, Tohoku University Graduate School of Medicine, 2-1 Seiryo-machi, Aoba-ku, Sendai 980-8575, Japan; 2 Division of Cell Proliferation, Tohoku University Graduate School of Medicine, 2-1 Seiryo-machi, Aoba-ku, Sendai 980-8575, Japan; 3 Department of Gene Expression Regulation, Institute of Development, Aging and Cancer, Tohoku University, 4-1 Seiryo-machi, Aoba-ku, Sendai 980-8575, Japan; 4 Division of Cancer Biology and Therapeutics, Miyagi Cancer Center Research Institute, Natori, Miyagi 981-1293, Japan; 5 Tohoku Medical-Megabank Organization, 2-1 Seiryo-machi, Aoba-ku, Sendai 980-8575, Japan

## Abstract

Nrf2 (NF-E2-related factor-2) transcription factor regulates oxidative/xenobiotic stress response and also represses inflammation. However, the mechanisms how Nrf2 alleviates inflammation are still unclear. Here, we demonstrate that Nrf2 interferes with lipopolysaccharide-induced transcriptional upregulation of proinflammatory cytokines, including IL-6 and IL-1β. Chromatin immunoprecipitation (ChIP)-seq and ChIP-qPCR analyses revealed that Nrf2 binds to the proximity of these genes in macrophages and inhibits RNA Pol II recruitment. Further, we found that Nrf2-mediated inhibition is independent of the Nrf2-binding motif and reactive oxygen species level. Murine inflammatory models further demonstrated that Nrf2 interferes with *IL6* induction and inflammatory phenotypes *in vivo*. Thus, contrary to the widely accepted view that Nrf2 suppresses inflammation through redox control, we demonstrate here that Nrf2 opposes transcriptional upregulation of proinflammatory cytokine genes. This study identifies Nrf2 as the upstream regulator of cytokine production and establishes a molecular basis for an Nrf2-mediated anti-inflammation approach.

## Abstract (web summary)

Nrf2 is a transcriptional activator of oxidative stress response genes. Here the authors show that Nrf2 binds to promoters of proinflammatory genes and interferes with their transcriptional upregulation in LPS-stimulated macrophages independently of its role in regulation of reactive oxygen species.

## Graphical abstract

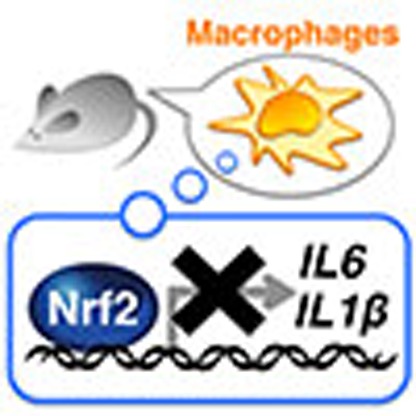

Controlling inflammation is critical in preventing various diseases, such as allergies, autoimmune diseases, cancer and metabolic syndromes^1^,^2^. Transcription factor Nrf2, which is essential for protection against oxidative/xenobiotic stresses, has been known to attenuate inflammation^3^,^4^. An *Nrf2*-deficiency causes an exacerbation of inflammation in a variety of murine models, such as sepsis, pleurisy and emphysema^5^,^6^,^7^,^8^, and also causes autoimmune phenotypes in some murine strains^9^,^10^. Consistently, Nrf2 activation in myeloid cells alleviates inflammation^11^. In human clinical studies, NRF2 inducer Tecfidera (dimethyl fumarate) has been approved for the treatment of multiple sclerosis^12^,^13^, in part based on its anti-inflammatory function. These observations indicate that Nrf2 is essential for the control of inflammation.

The regulatory system of Nrf2 activity has turned out as an attractive drug target due to its sophisticated mechanism^14^,^15^. Under unstressed conditions, Nrf2 is constitutively degraded through binding to Keap1 (Kelch-like ECH-associated protein 1), an adapter protein of E3 ubiquitin ligase. In the presence of oxidative and xenobiotic stresses, Nrf2 degradation is stalled, leading to a rapid accumulation of Nrf2. Nrf2 then translocates into the nucleus and forms a heterodimer with one of the small Maf proteins. Nrf2 binds to the regulatory regions of target genes to upregulate their transcription. Notably, a number of small molecules have been found to disrupt the Keap1-mediated degradation of Nrf2 and cause Nrf2 accumulation, and some of these molecules are approved or being developed for Nrf2-inducing therapy.

However, the mechanism underlying Nrf2-mediated anti-inflammation has not been clarified in detail. Since Nrf2 upregulates numerous antioxidant genes, elimination of reactive oxygen species (ROS) has been considered to be the molecular basis of Nrf2-mediated anti-inflammation. Indeed, recent reports suggest that elimination of ROS suppresses inflammation in *Nrf2*-deficient mice^8^,^16^. On the other hand, Nrf2 regulates the expression of macrophage-specific genes that are not categorized as anti-oxidative stress-response genes. For example, genes encoding MARCO, a scavenger receptor required for bacteria phagocytosis^17^, and CD36, a receptor for oxidized low-density lipoprotein related to atherosclerosis, are targets of Nrf2 (ref. 18). These insights imply that Nrf2 may also act as an anti-inflammatory regulator in a ROS-independent manner.

To address this issue, in this study we explored the Nrf2 target genes in the inflammatory state using proinflammatory (M1-) activated and anti-inflammatory (M2-) activated macrophages^19^. Microarray and Nrf2 Chromatin immunoprecipitation (ChIP) -seq analyses revealed that Nrf2 binds to the proximity of the proinflammatory cytokine genes, including *IL6* and *IL1b*, and inhibits lipopolysaccharide (LPS)-induced expression of these genes. This Nrf2-mediated transcriptional interference appears independent of ROS levels and independent of ARE (antioxidant responsive element). Notably, Nrf2 activation significantly disrupted the recruitment of RNA polymerase II (Pol II) to the *IL6* and *IL1b* loci. *In vivo* imaging analysis further revealed that Nrf2 activation inhibits *IL6* induction in murine inflammation model and alleviates inflammatory phenotypes. Thus, contrary to the current hypothesis that Nrf2 represses inflammation as a secondary consequence of anti-oxidation, these results unequivocally demonstrate that Nrf2 inhibits the induction of proinflammatory cytokine gene transcription. These findings indicate that Nrf2 is the key regulator for the two important cytoprotective pathways, anti-inflammation and anti-oxidation.

## Results

### Nrf2 inhibits proinflammatory cytokine gene expression

We first generated Nrf2-activated and Nrf2-depleted macrophages through conditional knockout of the *Keap1* gene in myeloid cells (KpCKO) and general knockout of the *Nrf2* gene (Nrf2-KO), respectively. The KpCKO mice were generated by crossing *Keap1*^*flox/flox*^ mice and *LysM*-Cre mice^20^,^21^. In KpCKO and Nrf2-KO mice, bone marrow-derived macrophages (BMDMs) were differentiated normally (Supplementary Fig. 1a,b), and comparable amounts of BMDMs were harvested from both lines of mice (Supplementary Fig. 1c), indicating that Nrf2 activation or Nrf2-depletion does not affect the differentiation and maturation of BMDMs. The number of neutrophils was also comparable among these genotypes (Supplementary Fig. 1d).

**Figure 1:**
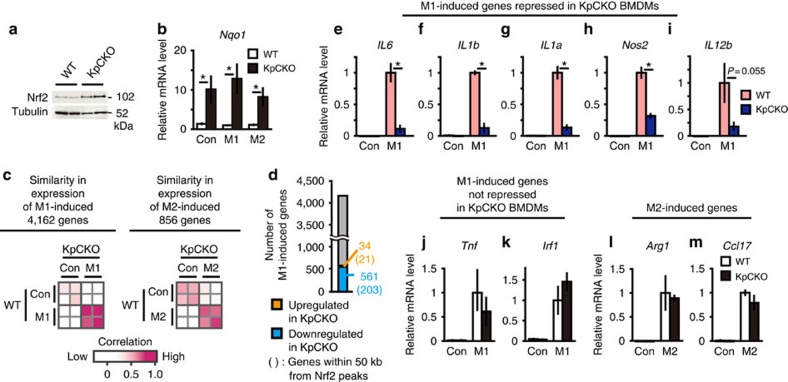
Genetic activation of Nrf2 inhibits M1 induction of proinflammatory cytokine genes. (**a**) Nrf2 protein expression in BMDMs from two independent WT and KpCKO mice, respectively. (**b**) Relative *Nqo1* gene expression. BMDMs from WT and KpCKO mice were either untreated (Con) or treated with M1 stimulation (M1, 5-ng ml^−1^ LPS and 10-ng ml^−1^ IFNγ) or M2 stimulation (M2, 10-ng ml^−1^ IL-4) for 6 h. (**c**) Similarity in gene expression of M1- and M2-induced genes between WT and KpCKO mice. With >2-fold change, 4,162 genes are upregulated by M1 stimulation (M1-induced genes), while 856 genes are upregulated by M2 stimulation (M2-induced genes), both in two independent WT mice. The expression pattern of M1- and M2-induced genes in WT and KpCKO BMDMs are compared to each other. The Pearson correlation coefficient for each comparison is depicted as colours from white (low) to magenta (high). (**d**) The number of M1-induced genes regulated by Nrf2. In 4,162 M1-induced genes, the number of genes upregulated or downregulated by *Keap1*-deficiency (with >2-fold change in KpCKO compared with WT in two independent BMDM cultures) are represented as orange and blue numbers, respectively. The numbers given in parentheses are the numbers of genes within 50 kb from Nrf2 peaks detected in the ChIP-seq analysis of M1-activated KpCKO BMDMs described below. (**e**–**m**) Relative expression of M1- and M2-induced genes in WT and KpCKO BMDMs. Expression of *IL6* (**e**), *IL1b* (**f**), *IL1a* (**g**), *Nos2* (**h**), *IL12b* (**i**), *Tnf* (**j**), *Irf1* (**k**), *Arg1* (**l**) and *Ccl17* (**m**) are presented. Data in **b**,**e**–**m** are mean±s.d. from three to four mice (*P<0.05, unpaired *t*-test).

In KpCKO mouse-derived BMDMs, Nrf2 accumulated significantly (Fig. 1a). This accumulation of Nrf2 induced the expression of *Nqo1* (*NAD(P)H quinone oxidoreductase 1*), a representative Nrf2 target gene. The extent of *Nqo1* induction was similar in control BMDMs, LPS/interferon (IFN) γ-treated M1-BMDMs, and IL-4-treated M2-BMDMs (Fig. 1b), indicating that accumulated Nrf2 functions equivalently in these BMDMs regardless of the polarization towards M1 and M2 macrophages.

Microarray analyses revealed that the expression of 4,162 and 856 genes was upregulated by the M1 and M2 stimulation, respectively. Expression profiles of these genes showed a strong correlation between KpCKO and wild-type (WT) BMDMs (Fig. 1c), indicating that the genetic Nrf2 activation does not affect the polarization of macrophages. Surprisingly, upregulation of a subset of M1-induced genes (561 genes) was specifically inhibited in KpCKO BMDMs (Fig. 1d, blue bar). These genes included *IL6, IL1b, IL1a, Nos2* and *IL12b*. The inhibition was confirmed by real-time quantitative PCR analyses (Fig. 1e–i). In contrast, expression levels of other M1-induced genes, such as *Tnf* and *Irf1*, and M2-induced genes, such as *Arg1* and *Ccl17*, were not changed in KpCKO BMDMs (Fig. 1j–m). These results indicate that Nrf2 inhibits the transcriptional induction of a subset of M1-induced genes.

### Nrf2 binds to the proximity of proinflammatory genes

**Figure 2:**
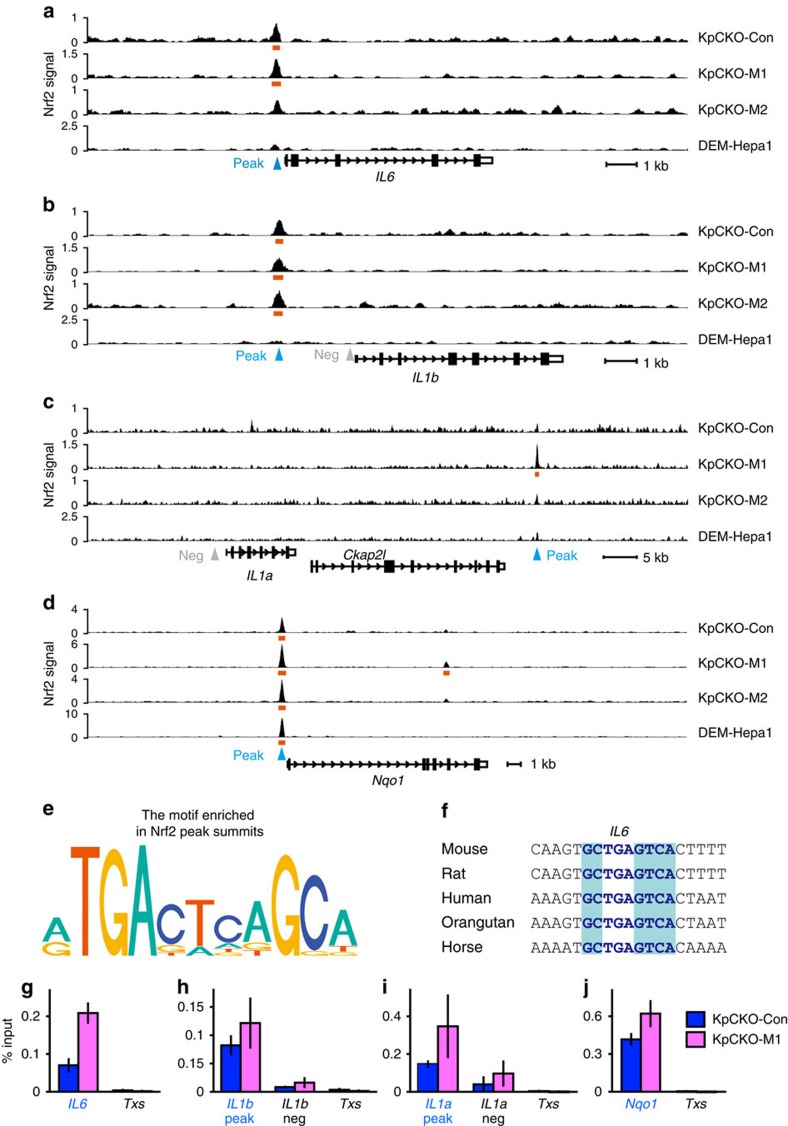
Nrf2 binding to the proximity of the proinflammatory cytokine genes. (**a**–**d**) Nrf2 ChIP-sequencing tracks of KpCKO BMDMs and DEM-treated Hepa1 cells. KpCKO BMDMs were either untreated (KpCKO-Con) or treated with M1 stimulation (KpCKO-M1, 5-ng ml^−1^ LPS and 10-ng ml^−1^ IFNγ) or M2 stimulation (KpCKO-M2, 10-ng ml^−1^ IL-4) for 4 h. Fragment pileup per million reads at the genomic loci proximal to the *IL6* (**a**), *IL1b* (**b**), *IL1a* (**c**) and *Nqo1* (**d**) genes are presented. DEM-Hapa1 shows the Nrf2 ChIP-seq track of DEM-treated Hepa1 cells^22^. Orange bars represent the peak regions. Primers for ChIP-qPCR described below were designed to amplify peak and non-peak (negative control) regions indicated by blue and grey triangles, respectively. (**e**) A consensus motif enriched in a ±25 bp region from Nrf2 peak summits in KpCKO-Con samples. (**f**) Phylogenetic conservation of ARE motifs in peak regions proximal to the *IL6*. Nucleotides identical to the core sequence of ARE, TGAG/CnnnGC, are in blue shades, and nucleotides in ARE identical to the murine sequences are depicted with the blue characters. (**g**–**j**) Nrf2 ChIP-qPCR analyses of KpCKO BMDMs. The Nrf2 signal was detected at the peak regions proximal to *IL6* (**g**), *IL1b* (**h**) and *IL1a* (**i**) genes (blue characters), similarly to the *Nqo1* promoter (**j**) used as a positive control. The intron of the *Txs* gene (*Txs*, **g**–**j**) and the non-peak regions proximal to the *IL1b* (**h**) and *IL1a* (**i**) genes were used as negative controls. Data in **g**–**j** are mean±s.d. from three mice (*P<0.05, unpaired *t*-test).

To examine whether Nrf2 directly binds to the M1-induced genes, we performed Nrf2 ChIP-seq analysis. In the M1-induced gene loci, Nrf2 binding was observed in the upstream regions of the *IL6* and *IL1b* genes (Fig. 2a,b). Nrf2 binding was also detected in the downstream region of the *IL1a* gene (Fig. 2c), but this Nrf2-binding peak was far away from the *IL1a* gene TSS (transcription start site), so that the site may not be involved in the *IL1a* gene regulation. The expression of *Ckap2l* gene, located between the *IL1a* gene and the Nrf2 peak, was not changed upon both Nrf2 activation and M1 stimulation (Supplementary Fig. 2a).

Notably, Nrf2-binding peaks near the *IL6* and *IL1b* genes were not observed in our previous Nrf2 ChIP-seq analysis with Hepa1 cells^22^ (Fig. 2a,b). This is in clear contrast to the Nrf2 binding near representative anti-oxidative Nrf2 target gene loci, *Nqo1* (Fig. 2d) and *Hmox1* (Supplementary Fig. 2b), in which Nrf2 binding was observed both in BMDMs and Hepa1 cells. In the Nrf2 peaks, ARE (TGAG/CnnnGC)^23^,^24^ was enriched in their peak summits, consistent with the previous Nrf2 ChIP-seq study^22^ (Fig. 2e). When we examined phylogenetic conservation of ARE sequences, we found that the consensus motif is conserved among various mammalian genomes at the Nrf2 peak summit proximal to the *IL6* gene (Fig. 2f). On the other hand, Nrf2-binding regions proximal to *IL1b* and *IL1a* genes did not contain conserved TGAG/CnnnGC motif at the peak summits. Therefore, we surmise that Nrf2 is recruited through the association with other proteins rather than direct binding to the TGAG/CnnnGC motif or ARE, at least at the *IL1b* and *IL1a* loci. The ARE localized in the proximity of *IL6* gene has been reported to upregulate *IL6* expression in hepatocytes^25^, suggesting that Nrf2 binds to the *IL6* gene also in hepatic cells, but activates gene expression, in contrast to the repression in macrophages.

Binding of Nrf2 was further confirmed by ChIP-qPCR analyses of BMDMs derived from KpCKO mice (Fig. 2g–j). In the peak regions proximal to the *IL6*, *IL1b, IL1a* and *Nqo1* genes, the Nrf2-binding signal was higher than the negative control loci, that is, *Thromboxane synthase* gene (*Txs*) intron and the non-peak regions proximal to the *IL1b* and *IL1a* genes (Fig. 2g–j). Nrf2 binding to proinflammatory cytokine loci was observed in both untreated and M1-activated cells, although the most prominent was observed in M1-stimulated BMDMs at the *IL6* and *IL1a* loci.

### Nrf2 negatively regulates a subset of M1-induced genes

**Figure 3:**
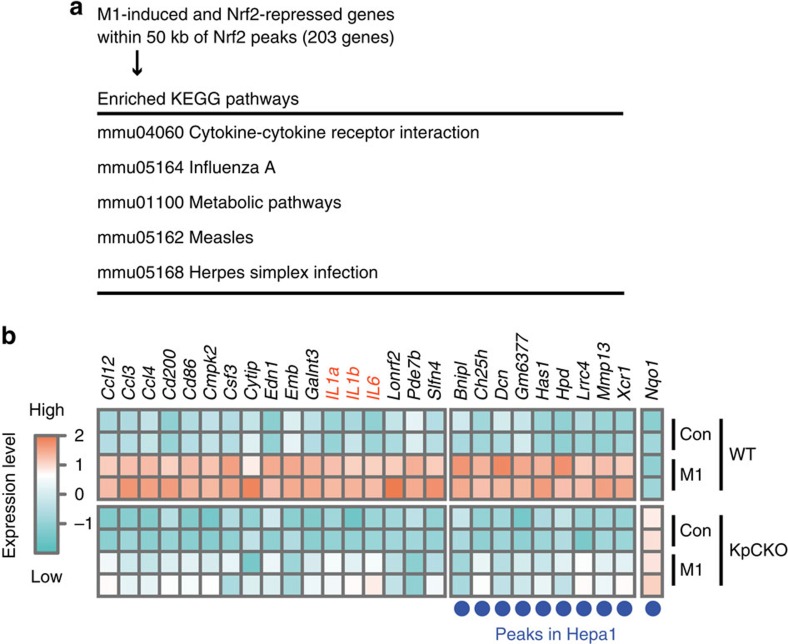
Microarray and ChIP-seq analyses of *Keap1*-deficient BMDMs. (**a**) KEGG pathway analysis on M1-induced and Nrf2-repressed genes within 50 kb of Nrf2 peaks detected in KpCKO-M1 samples. The top five pathways are presented. (**b**) Expression profiles of representative M1-induced genes repressed by Nrf2. BMDMs from two independent WT and KpCKO mice were either untreated (Con) or treated with M1 stimulation (M1, 5-ng ml^−1^ LPS and 10-ng ml^−1^ IFNγ) for 6 h and rendered for microarray analysis. Each row represents the mRNA expression level in the corresponding mice depicted as colours from turquoise (low) to coral (high). Each column represents one of 26 genes located within 50 kb of the Nrf2-binding peaks in KpCKO-M1 BMDMs, whose expression was induced by M1 stimulation in WT BMDMs (fold change >2) and repressed by *Keap1*-deficiency in M1-stimulated BMDMs (fold change <−4). The expression of *Nqo1* is displayed to show the expression pattern of typical Nrf2 target genes. Blue dots under the figure represent genes within 50 kb of the Nrf2-binding peaks obtained in DEM-treated Hepa1 cells. Note that the inflammation-related genes are accumulated in the left group of genes, which is inhibited by Nrf2 in a macrophage-specific manner.

We then conducted an association analysis of the microarray data and ChIP-seq data of M1-induced macrophage genes. The results indicate that of the 561 genes repressed by Nrf2, 203 genes had Nrf2-binding peaks in their proximal regions (Fig. 1d). We also conducted Kyoto Encyclopedia of Genes and Genomes (KEGG) pathway analysis^26^ for these 203 genes and found that several important pathways were associated with these genes (Fig. 3a). The pathways related to the inflammatory diseases were enriched in these genes, supporting the notion that Nrf2 contributes to the resolution of inflammation. These 203 genes displayed expression profiles distinct from those of typical Nrf2 target genes, as the Nrf2 activation in KpCKO mice strongly repressed the expression of these 203 genes. Importantly, 132 out of these 203 Nrf2-repressed genes harboured macrophage-specific Nrf2-binding peaks in their proximal regions, as was the cases for the *IL6*, *IL1b* and *IL1a* genes (Fig. 3b). In contrast, some of the 203 genes retained Nrf2-binding peaks both in M1-macrophage and Hepa1 cells (Fig. 3b, blue dots). Collectively, these results imply that Nrf2 directly inhibits the M1-induced expression of various inflammation-related genes through binding to their proximal regulatory regions.

### Nrf2-dependent inhibition of proinflammatory gene expression

**Figure 4:**
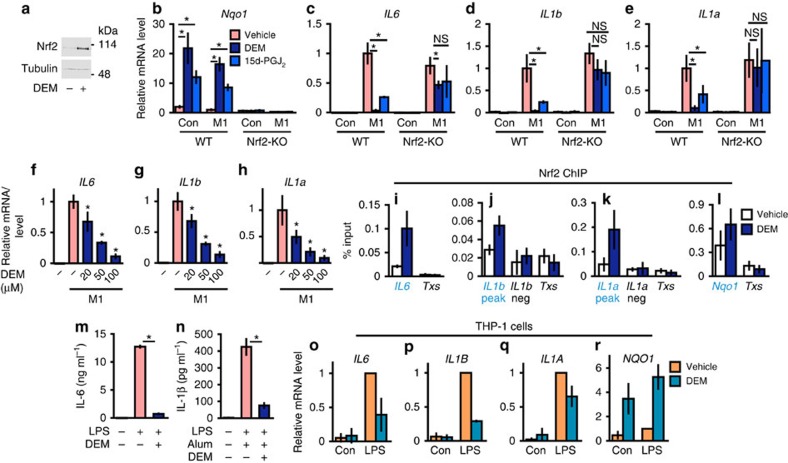
Inhibition of M1-induced proinflammatory cytokine genes by chemical Nrf2 inducers. (**a**) Nrf2 protein accumulation in the WT BMDMs treated with 100-μM DEM for 4 h (representative data, *n*=4). (**b**–**e**) Inhibition of proinflammatory gene expression by Nrf2 inducers. BMDMs from WT and Nrf2-KO mice were untreated (Con) or M1-stimulated (M1, 5-ng ml^−1^ LPS and 10-ng ml^−1^ IFNγ) in the presence of 100-μM DEM (DEM), 10-μM 15d-PGJ_2_ (15d-PGJ_2_) or vehicle for 6 h. Relative expressions of *Nqo1* (**b**), *IL6* (**c**), *IL1b* (**d**) and *IL1a* (**e**) genes were examined. Note that the inhibition of *IL6*, *IL1b* and *IL1a* genes and *Nqo1*-induction are cancelled by *Nrf2*-knockout. (**f**–**h**) Dose-dependent inhibition of proinflammatory genes by the Nrf2 inducer. Relative expressions of *IL6* (**f**), *IL1b* (**g**) and *IL1a* (**h**) genes after 6 h of M1-stimulation in the presence of increasing doses of DEM. *P<0.05 (unpaired *t*-test) against vehicle-treated M1-BMDMs. (**i**–**l**) Nrf2 ChIP-qPCR of DEM-treated WT BMDMs. WT BMDMs were treated with 100-μM DEM for 4 h. Nrf2 signal was detected at the peak regions proximal to the *IL6* (**i**), *IL1b* (**j**), *IL1a* (**k**) and *Nqo1* (**l**) genes (blue triangles in Fig. 2a–d). The *Txs* intron (*Txs*, **i**–**l**) and the non-peak regions near the *IL1b* (**j**) and *IL1a* (**k**) genes were used as negative controls (grey triangles in Fig. 2b,c). (**m**,**n**) The concentration of IL-6 (**m**) and IL-1β (**n**) secreted in the culture media. BMDMs were stimulated with 5-ng ml^−1^ LPS and 100-μg ml^−1^ alum in the presence of 100-μM DEM or vehicle for 24 h. (**o**–**r**) Inhibition of proinflammatory genes by the Nrf2 inducer in human cells. THP-1 cells were untreated (Con) or LPS-treated (LPS, 5-ng ml^−1^ LPS) in the presence of 100-μM DEM or vehicle for 6 h. Relative expressions of *IL6* (**o**), *IL1B* (**p**), *IL1A* (**q**) and *NQO1* (**r**) are presented. Data are mean±s.d. from three mice (**b**–**n**) or three individual samplings (**o**–**r**). *P<0.05, unpaired *t*-test; NS, not significant.

To ascertain Nrf2 dependency of the inhibition of proinflammatory cytokine gene induction, we tested the consequences of chemical induction of Nrf2. For the pharmacological activation of Nrf2, we used two small molecule inducers: diethylmaleate (DEM), an electrophilic reagent, and 15-deoxy-Δ^12,14^-prostaglandin *J*_2_ (15d-PGJ_2_), an endogenous electrophilic lipid that is naturally generated in macrophages^5^. These inducers accumulated Nrf2 (Fig. 4a) and significantly upregulated the *Nqo1* expression in an Nrf2-dependent manner (Fig. 4b).

Showing a stark contrast, treatment with the inducers markedly inhibited the transcriptional induction of *IL6*, *IL1b* and *IL1a* in M1-induced macrophages (Fig. 4c–e), as was the case for the genetic activation of Nrf2 in KpCKO. The inhibition was cancelled in Nrf2-KO BMDMs (Fig. 4c–e), indicating direct contribution of Nrf2. DEM inhibited the *IL6*, *IL1b* and *IL1a* gene expression dose-dependently (Fig. 4f–h).

We then examined Nrf2 binding to the proximity of the *IL6*, *IL1b* and *IL1a* genes upon DEM treatment. The ChIP-qPCR results clearly indicated that Nrf2 binds to the proximity of these inflammatory cytokine genes (Fig. 4i–k) and also to the *Nqo1* gene (Fig. 4l) in the DEM-treated BMDMs, even without LPS treatment. Notably, the DEM treatment significantly inhibited the LPS-induced and LPS/alum-induced secretion of IL-6 (Fig. 4m) and IL-1β (Fig. 4n) into the culture media, respectively. These results clearly demonstrate that Nrf2 binds to the regulatory regions of these inflammatory cytokine genes and reduces these cytokine productions.

We then examined whether this negative regulation of the inflammatory cytokine genes by Nrf2 is also operative in human macrophage-related cells. To this end, we exploited human monocytic THP-1 cells^27^. Showing very good agreement with the results in mice, NRF2 activation by DEM inhibited the LPS-induced transcriptions of the *IL6*, *IL1B* and *IL1A* genes, but activated the *NQO1* gene expression in the THP-1 cells (Fig. 4o–r). These results indicate that the Nrf2-mediated inhibition of proinflammatory cytokine gene induction is conserved in human monocytic cells. Thus, consistent with the results of the genetic analyses, the pharmacological induction of Nrf2 also markedly inhibited the transcription of proinflammatory cytokine genes both in murine and human cells.

### Nrf2-mediated inhibition is independent of redox control

**Figure 5:**
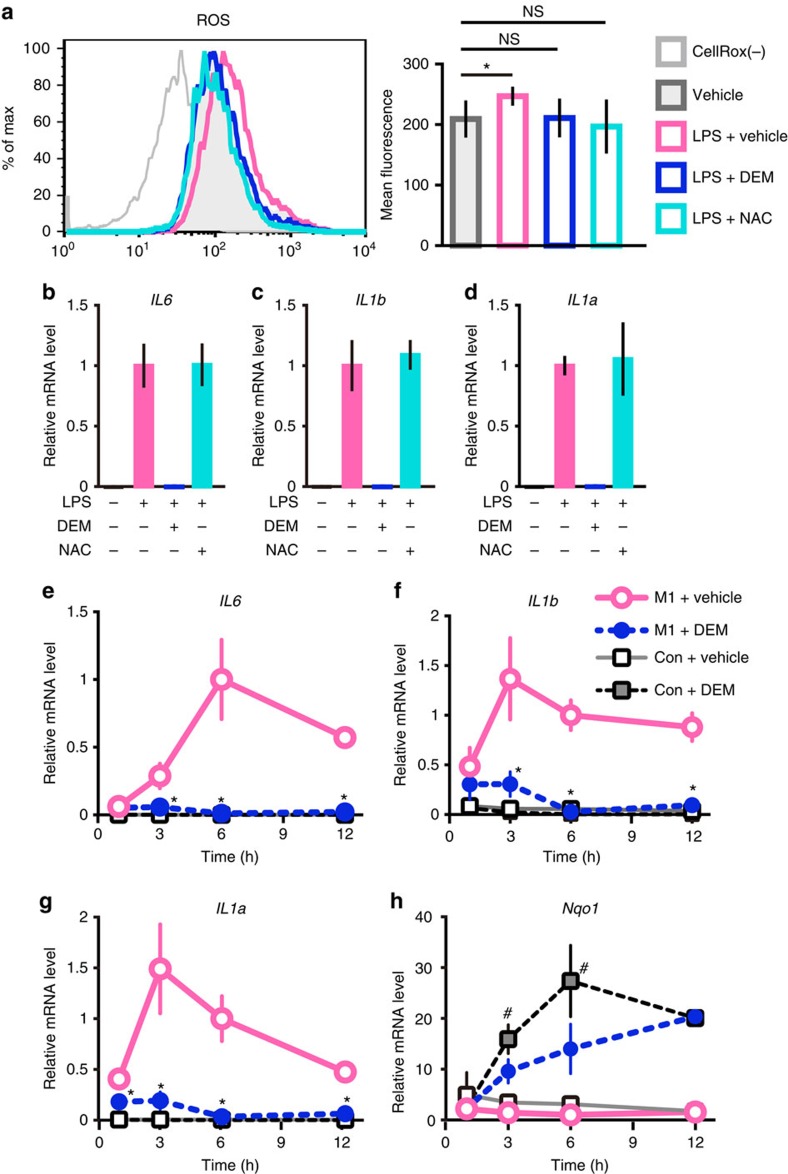
Nrf2-mediated inhibitions of proinflammatory cytokine genes are independent of ROS elimination. (**a**) Evaluation of the ROS level in DEM- or NAC-treated BMDMs. WT BMDMs were stimulated with 5-ng ml^−1^ LPS or vehicle, with or without 100-μM DEM or 1-mM NAC, for 4 h. The ROS level was evaluated by fluorescence after treatment with CellROX reagent during the last 30 min of 4 h stimulation. Data are mean±s.d. from four mice (*P<0.05, paired *t*-test). (**b**–**d**) Evaluation of the ROS-mediated effect. The relative expressions of *IL6* (**b**), *IL1b* (**c**) and *IL1a* (**d**) were examined by RT-qPCR after the same stimulations as (**a**). Note that NAC-treatment does not affect these cytokine mRNA expressions. (**e**–**h**) Time-course change of inflammatory cytokine gene expression after the chemical Nrf2 inducer treatment. Relative mRNA expression of *IL6* (**e**), *IL1b* (**f**), *IL1a* (**g**) and *Nqo1* (**h**) in WT BMDMs were examined at indicated times from the start of M1 stimulation in the presence of 100-μM DEM or vehicle. DEM and vehicle were added in the culture media at the same time as the M1 stimulus. The value of M1+Vehicle at 6 h is set to 1. *P<0.05 (unpaired *t*-test) against M1+Vehicle; ^#^*P*<0.05 (unpaired *t*-test) against Con+Vehicle. Note that DEM inhibits the expression of the inflammatory cytokine mRNAs, but induces the expression of *Nqo1* mRNA.

Since Nrf2 regulates expression of numerous genes that participate in the control of ROS, we examined whether the Nrf2-mediated inhibition of proinflammatory cytokine gene induction depends on the ROS levels. For this purpose, we treated BMDMs with antioxidant *N*-acetyl cysteine (NAC), a precursor of glutathione. LPS treatment significantly upregulated the ROS levels, while both of DEM- and NAC-treatment reduced ROS to the basal level (Fig. 5a). However, the NAC-treatment did not affect the LPS-induced transcription of the *IL6*, *IL1b* and *IL1a* genes (Fig. 5b–d), strongly supporting our contention that decrease of the ROS level does not provoke the inhibition of proinflammatory cytokine genes.

To gain further insights into the relation of anti-inflammation and activation of Nrf2, we next examined the temporal order of inhibition and activation of Nrf2 target genes. After the M1 stimulation, the expression of *IL6, IL1b* and *IL1a* mRNAs was quickly induced, as early as 3 h after the stimulation (Fig. 5e–g). Of note, simultaneous treatment with the Nrf2 inducer DEM almost completely suppressed the proinflammatory gene induction from the early time-points (Fig. 5e–g). This immediate suppression occurred almost at the same timing as the DEM-mediated induction of the Nrf2 target genes, that is, *Nqo1* (Fig. 5h), *Hmox1* and *Gclc* (Supplementary Fig. 3a,b). These results indicate that the transcriptional inhibition is a primary event provoked by Nrf2, rather than a secondary consequence of the elimination of ROS through the production of antioxidant enzymes and proteins.

To clarify whether this Nrf2-mediated inhibition depends on the order of LPS treatment and Nrf2 activation by DEM, we changed the timing of the LPS and DEM additions. In an experiment shown in Supplementary Fig. 4a, macrophages were first treated with DEM for 3 h and then treated with LPS for an additional 3 h, while in an experiment shown in Supplementary Fig. 4b, the order was switched. The results clearly showed that DEM inhibited the proinflammatory cytokine gene expression regardless of the order of LPS and DEM treatment.

### Nrf2 inhibits recruitment of RNA polymerase II

**Figure 6:**
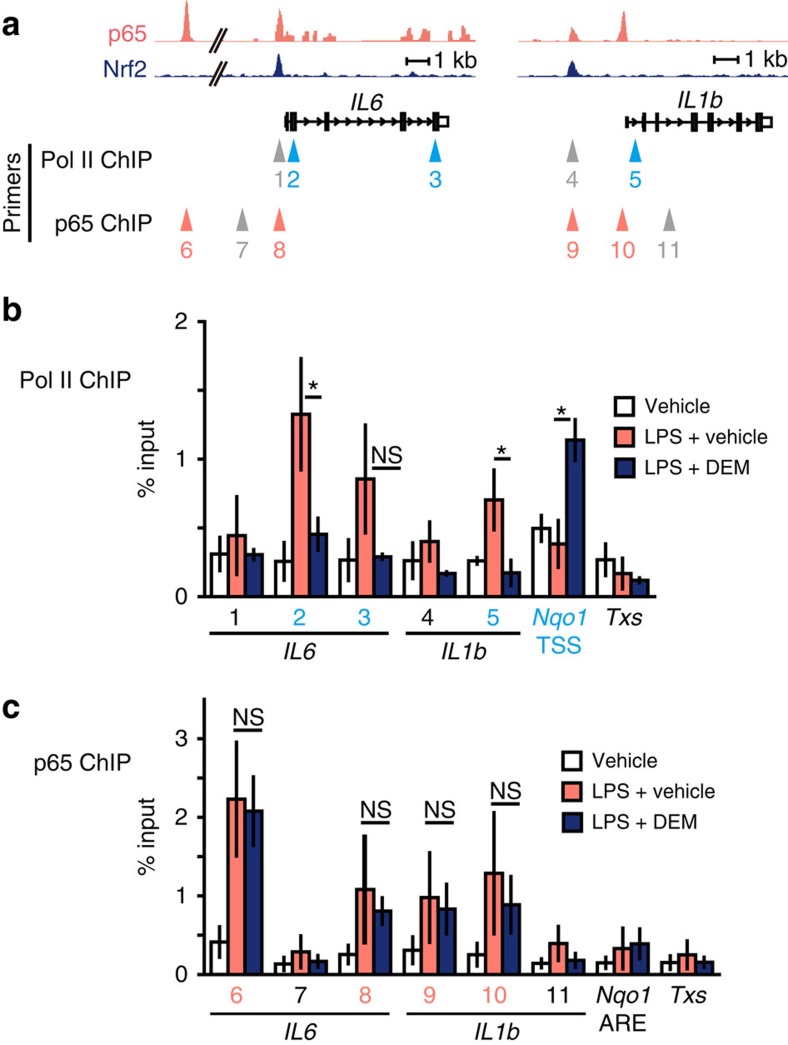
Nrf2 inhibits the recruitment of RNA Pol II on the proinflammatory cytokine gene loci without affecting the NF-κB p65 recruitment. (**a**) ChIP-sequencing track specific for p65 at the *IL6* and *IL1b* loci^29^ and Nrf2 ChIP-sequencing track of KpCKO-M1 BMDMs depicted in Fig. 2. Primers for the ChIP-qPCR were designed to amplify the regions indicated with triangles. Grey triangles represent the negative control regions. (**b**) Pol II ChIP-qPCR analyses of WT BMDMs. BMDMs were stimulated with 5-ng ml^−1^ LPS, in the presence of 100-μM DEM or vehicle, for 4 h. Numbers under the bar graph represent the peak or non-peak regions depicted in (**a**). The binding of Pol II was upregulated in LPS-treated cells at the TSS of *IL6* (2), the gene body of *IL6* (3) and the TSS of the *IL1b* gene (5), similar to the TSS of the *Nqo1* gene in LPS+DEM-treated cells used as a positive control (*Nqo1* TSS). The intron of the *Txs* gene and the promoter regions of *IL6* (1) and *IL1b* (4) were used as negative controls. (**c**) p65 ChIP-qPCR analyses of WT BMDMs. BMDMs were treated with LPS and/or DEM as in (**b**). The binding of p65 was upregulated in LPS-treated cells at the p65-binding sites proximal to *IL6* (8) and *IL1b* (9, 10), similarly to the 60 kb upstream site of the *IL6* gene (6) used as a positive control. The *Txs* intron, the Nrf2-binding site near *Nqo1* gene (*Nqo1* ARE) and the regions without p65 binding near the *IL6* (7) and *IL1b* (11) genes were used as negative controls. Data are mean±s.d. from three mice (*P<0.05, unpaired *t*-test; NS, not significant).

In contrast to the widely accepted view that Nrf2 suppresses the inflammation through ROS elimination, our results thus far suggest that Nrf2 regulates the inflammation through transcriptional inhibition of proinflammatory cytokine *IL6* and *IL1b* genes. To determine mechanisms how Nrf2 inhibits the induction of a subset of inflammatory cytokine genes, we examined changes that Nrf2 elicits in the transcription of these genes. We first conducted ChIP-qPCR analyses exploiting BMDMs and a Pol II-specific antibody to dissect the transcription initiation step. The results revealed that LPS-induced binding of Pol II to the proximity of TSSs of the *IL6* and *IL1b* genes was significantly inhibited by concomitant DEM treatment (Fig. 6a,b), indicating that Nrf2 inhibits the transcriptional initiation of these proinflammatory cytokine genes through the interference with Pol II recruitment to the TSSs.

Since expression of many cytokine genes are regulated at the post-transcriptional level^28^, we also examined stability of the cytokine mRNAs. However, the Nrf2 activation did not affect substantially the mRNA stability of *IL6*, *IL1b* and *IL1a* (Supplementary Fig. 5a). We also assessed pre-mRNA syntheses from the *IL6*, *IL1b* and *IL1a* genes and found that Nrf2 inhibited the pre-mRNA syntheses, and that the magnitudes of the inhibition were practically equivalent to those of the mature mRNA reduction (Supplementary Fig. 5b). These results thus indicate that Nrf2 regulates *IL6* and *IL1b* gene expression at the transcription initiation step.

It has been postulated that Pol II is recruited to the TSSs of inflammation-related genes through association with a group of inflammation-responding transcription factors^19^, and Nrf2 activation has been reported to inhibit NF-κB signal^11^. In our survey of various ChIP-seq data, we found that transcription factors required for inflammation-induced expression of *IL6* gene, p65 (NF-κB) (ref. 29), C/EBPβ (ref. 30) and c-Jun (AP-1) (ref. 31), bind in close proximity of Nrf2-binding sites (Fig. 6a and Supplementary Fig. 6a). Therefore, we hypothesized that Nrf2 might inhibit the recruitment of these transcription factors. We tested this hypothesis by conducting ChIP-qPCR analyses exploiting BMDMs and p65-specific antibody and found that LPS markedly enhanced the recruitment of p65 to the proximity of the *IL6* and *IL1b* genes. However, the recruitment of p65 was unchanged by the DEM treatment, indicating that NF-κB was recruited normally to the *IL6* and *IL1b* gene regulatory regions even under the Nrf2 activation (Fig. 6c). Similarly, recruitment of C/EBPβ was also unchanged upon the Nrf2 activation (Supplementary Fig. 6b).

Furthermore, while LPS treatment provoked certain expected changes in the histone modifications, including decrease in tri-methylation of histone H3 at lysine 27 (H3K27me3) and increase in H3K27 acetylation (H3K27Ac) and H3K4 tri-methylation (H3K4me3), Nrf2 activation by DEM treatment did not change these modification substantially (Supplementary Fig. 6c–e). We also found that Nrf2 normally associates with co-activator CBP (ref. 32) and the components of Mediator complex^33^, MED16 and MED24 (Supplementary Fig. 7) in the presence of DEM. These co-activator associations did not decrease, or rather increased, in the LPS-treated cells compared with LPS-untreated cells. Our results thus demonstrate that Nrf2 inhibits the transcription initiation and Pol II recruitment to the *IL6* and *IL1b* TSSs without changing the recruitment of proinflammatory transcription factors. Changes in the co-activators and the histone modifications wait for further extensive and elaborate analyses.

### Nrf2-mediated inhibition is Nrf2 binding motif-independent

**Figure 7:**
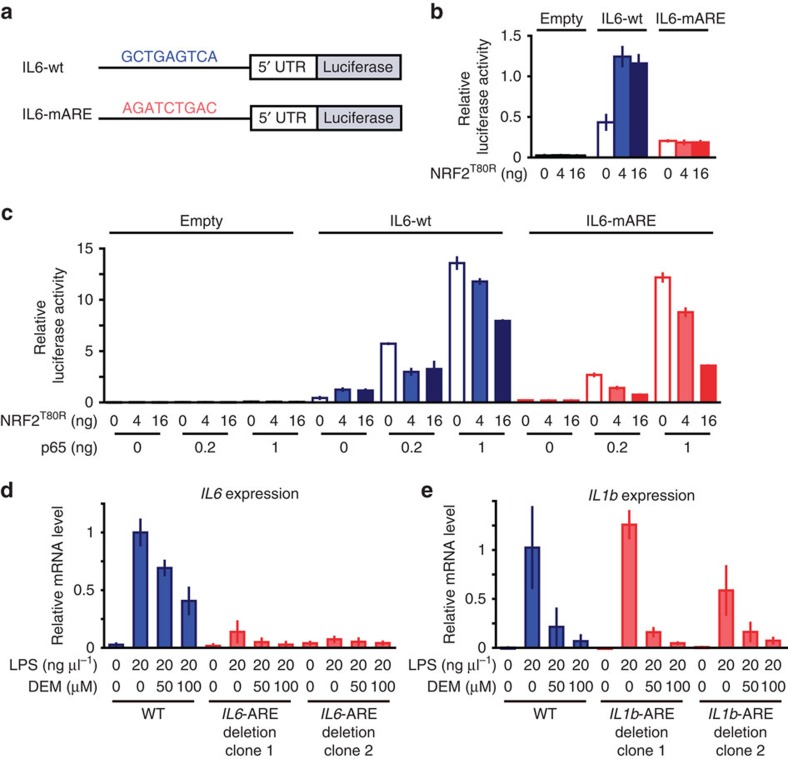
Nrf2-mediated inhibition of proinflammatory cytokine genes is independent of ARE in upstream regulatory region. (**a**) The schema of luciferase reporter vectors. Approximately 1 kb region from the translation start site of *IL6* gene was conjugated to the translation start site of NanoLuc reporter vector (IL6-wt). The ARE motif in WT *IL6* locus, GCTGAGTCA (Fig. 2f), is mutated to AGATCTGAC in the ARE-mutant reporter vector (IL6-mARE). (**b**) Relative luciferase activity of IL6-wt and IL6-mARE reporter vectors in the Raw264.7 cells transfected with constitutive active form of NRF2 (NRF2^T80R^). (**c**) Relative luciferase activity of IL6-wt and IL6-mARE reporter vectors in the Raw264.7 cells transfected with NRF2^T80R^ and p65. (**d**,**e**) Relative *IL6* and *IL1b* mRNA expressions in the parental Raw264.7 cells and derived cells in which Nrf2-binding region proximal to the gene is deleted. We deleted the ARE-containing regulatory regions from the *IL6* (**d**) and *IL1b* (**e**) genes (Supplementary Fig. 8) by means of the CRISPR/Cas9 genome-editing technology. Cells were treated with LPS and/or DEM for 6 h. The value of LPS-stimulated parental Raw264.7 cells is set to 1 (**d**,**e**).

To further delineate the mechanisms how Nrf2 operates the inhibition of proinflammatory cytokine gene induction, we next conducted a luciferase reporter assay using an ∼1 kb region upstream of the *IL6* gene that includes ARE. In IL6-wt construct, upstream sequence of the *IL6* gene was ligated to a luciferase expression vector, while in IL6-mARE construct, ARE motif in the region was mutated (Fig. 7a). Co-transfection of constitutive active form of NRF2 (NRF2^T80R^) in 293T cells moderately upregulated the luciferase reporter expression from IL6-wt, but not from IL6-mARE (Fig. 7b), indicating that Nrf2 functions as a transcriptional activator even at the *IL6* locus. This result is consistent with the previous report using hepatoma cells^25^. In contrast, co-transfection of p65 markedly elevated the luciferase reporter expression, indicating that NF-κB acts as the primary inducer of the *IL6* gene. Importantly, NRF2^T80R^ inhibited the reporter expression in a dose dependent manner and this inhibition was observed even in the absence of ARE (Fig. 7c).

These results suggest that Nrf2 inhibits the *IL6* gene expression by interfering with the p65-mediated transcriptional activation of the gene. Given that inflammation-responding transcription factors bind to the close proximity of Nrf2-binding region (Fig. 6a,c and Supplementary Fig. 6a,b), Nrf2 may interfere with the function of these transcription factors, such as p65, and/or co-factors required for inflammation-induced transcription.

To ascertain the finding that ARE might not be required for the Nrf2-mediated inhibition of *IL6* induction, we deleted the Nrf2-binding regions including the ARE in macrophage-derived Raw264.7 cells using CRISPR/Cas9 system (Supplementary Fig. 8a,b). We analysed two clones, in which the centre region of ChIP Nrf2-binding peak including the ARE was deleted. As the proinflammatory transcription factor-binding sites are located in the close proximity of the ARE, there is a possibility that the deletion of this region may disrupt the recruitment of transcription factors required for induction of *IL6* gene expression. Indeed this was the case and the deletion abolished the LPS-induced expression of *IL6* gene (Fig. 7d). Thus, our aim to evaluate the ARE contribution to the Nrf2-mediated inhibition cannot be accomplished.

In contrast, deletion of Nrf2-binding region proximal to the *IL1b* gene kept the LPS-induction, perhaps due to preservation of the binding sites of activating transcription factors (Fig. 7e). Importantly, the DEM-mediated induction of Nrf2 nicely inhibited the LPS-mediated upregulation of *IL1b* gene expression, and the deletion of Nrf2-binding region did not affect the Nrf2-mediated inhibition of the *IL1b* induction, indicating that Nrf2-binding region is not essential for the *IL1b* inhibition, as is the case for ARE in the *IL6* upstream region. One plausible explanation for these observations is that Nrf2 may be recruited to the *IL6* and *IL1b* gene regulatory regions through the association with other transcription factors or co-factors.

### Nrf2 inhibits *IL6* induction in murine inflammatory models

**Figure 8:**
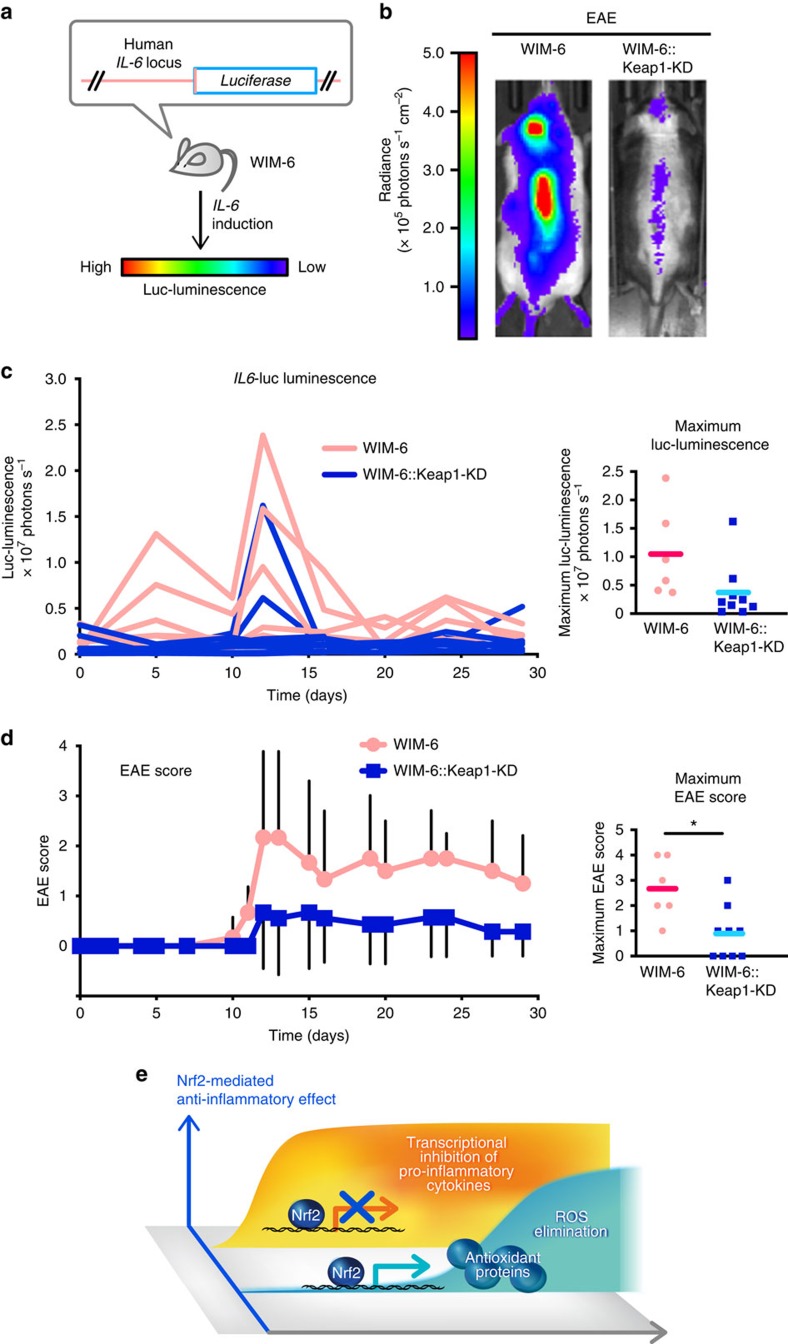
Nrf2 inhibits *IL6* expression and alleviates inflammation *in vivo*. (**a**) The schema of *in vivo* monitoring of *IL6* expression using WIM-6 mice^34^. WIM-6 mice express the luciferase gene under the control of the human *IL6* promoter, and *IL6*-inducing stimuli lead to the emission of luciferase luminescence in WIM-6 mice. *IL6*-luc luminescence intensity in the following figure is depicted as colours from red (high) to blue (low). (**b**,**c**) *In vivo* monitoring of *IL6* expression in the EAE model. WIM-6 mice with WT *Keap1* (WIM-6) or with *Keap1*-knockdown (WIM-6::Keap1-KD) on the C57BL/6 background were subjected to EAE induction. (**b**) Representative images of *IL6*-luc luminescence of WIM-6 mouse (left) and WIM-6::Keap1-KD mouse (right) at day 12 are shown. (**c**) Time course of the *IL6*-luc luminescence intensity in each mouse from WIM-6 group (red line, *n*=6) and WIM-6::Keap1-KD group (blue line, *n*=9) are shown in left panel. The maximum *IL6*-luc luminescence in the observation period is shown in right panel. Red and blue bars represent the means of the maximum *IL6*-luc luminescence from WIM-6 and WIM-6::Keap1-KD mice, respectively. (**d**) Clinical EAE scores in the same experiment as in **c**. The mean±s.d. of EAE score (left) and the maximum EAE score in the observation period (right) are shown. Note that the EAE score was significantly repressed in WIM-6::Keap1-KD mice. Red and blue bars represent the mean of the maximum EAE score. *P<0.05, unpaired *t*-test. (**e**) The illustration shows the schema of the Nrf2-mediated anti-inflammatory effect. Nrf2 binds to the proximity of inflammatory cytokine genes, including *IL6* and *IL1b*, and inhibits their transcription. At the same time Nrf2 upregulates expression of genes coding antioxidant proteins. These antioxidant proteins eliminate ROS and subsequently contribute to the anti-inflammation.

Finally, we utilized the whole mount *in vivo* monitoring system with *hIL6*-luc reporter (WIM-6) to verify the Nrf2-mediated inhibition of *IL6* gene induction *in vivo* (Fig. 8a). WIM-6 mice harbour firefly luciferase transgenes, which is homologously recombined to the human *IL6* gene in a bacterial artificial chromosome vector^34^. Thus, WIM-6 mice emit luminescence under the *IL6*-inducing stimuli. In this study, we executed an experimental autoimmune encephalomyelitis (EAE) model known as a murine model of multiple sclerosis, because IL-6 has been shown to be responsible for EAE development^35^. After immunization with myelin oligodendrocyte glycoprotein, WIM-6 mice on the C57BL/6 background exhibited intense *IL6*-luc luminescence in their brains and spinal cords due to autoimmunity against the myelin component (Fig. 8b, left). Time course of the increase in luminescence showed a maximum emission of luminescence after day 10 (Fig. 8c), simultaneously with an increase in the EAE score (Fig. 8d).

In contrast, when we activated Nrf2 in WIM-6 mice through concomitant knockdown of *Keap1* (Keap1-KD), the *IL6*-luc luminescence upon EAE induction was remarkably reduced (Fig. 8b, right) throughout the experimental period (Fig. 8c). The maximum *IL6*-luc luminescence in the observation period was lower in Keap1-KD::WIM-6 mice than in WT Keap1::WIM-6 mice, although a few Keap1-KD mice showed the increase of *IL6*-luc luminescence. The EAE clinical score was also reduced in Keap1-KD::WIM-6 mice (Fig. 8d). The maximum EAE score in the observation period was significantly reduced in Keap1-KD::WIM-6 mice compared with WT Keap1::WIM-6 mice (Fig. 8d, right). We also conducted similar analyses exploiting WIM-6 mice on the ICR/CD1 background, which were preferable for *in vivo* imaging due to their white fur coat, but they were low responders to EAE. Showing very good agreement with the results of C57BL/6 mice, Keap1-KD::WIM-6 mice on the ICR/CD1 background exhibited lower *IL6*-luc luminescence (Supplementary Fig. 9a–c) and EAE score (Supplementary Fig. 9d,e) than WT Keap1::WIM-6 mice did.

To further confirm the Nrf2-medited inhibition of *IL6* gene induction *in vivo*, we utilized another inflammation model, that is, *Staphylococcus aureus* infection. Upon infection with *S. aureus*, WIM-6 mice with Nrf2 knockout showed much more intense *IL6*-luc luminescence on their back than the WIM-6 mice with normal Nrf2 expression showed (Supplementary Fig. 10a,b). This experiment was executed by using the WIM-6 mice on the ICR/CD1 background. These results clearly indicate that Nrf2 inhibits the *IL6* expression and alleviates inflammatory symptoms *in vivo*.

## Discussion

While contributions of Nrf2 to anti-inflammation have been recognized, molecular basis of the Nrf2 function has long been obscure. In this study, two important points have been clarified. First, we discovered that Nrf2 inhibits the LPS-induced expression of proinflammatory cytokine genes, including *IL6* and *IL1b*, through the ROS-independent transcriptional inhibition. We believe that this finding will be a turning point in understanding the Nrf2 function related to inflammation, because we previously assumed that Nrf2 function was limited to oxidative stress control and that anti-inflammation was merely a consequence of ROS elimination. In contrast to this dogma, this study clearly unravels that Nrf2 is the key regulator of proinflammatory cytokine expression as well as intracellular ROS, as summarized in Fig. 8e. Second, while Nrf2 has been recognized as an activating transcription factor, our data clarify the presence of transcription-inhibiting function of Nrf2. The binding of Nrf2 in close proximity of the *IL6* and *IL1b* genes implies that Nrf2 inhibits transcription through direct DNA binding. However, our analyses indicate that Nrf2-mediated inhibition of the inflammatory cytokine gene expression in M1 macrophages is ARE-independent. Furthermore, Nrf2 inhibits Pol II recruitment to the TSSs of the *IL6* and *IL1b* genes without affecting the NF-κB recruitment required for the expression of *IL6* and *IL1b* genes. Although precise elucidation of the molecular basis how Nrf2 elicits the transcriptional inhibition is rather complicated and still needs to be elucidated, our present findings support the notion that the Nrf2 regulates negatively the target genes that encode inflammatory cytokines.

Mechanisms how Nrf2 inhibits the transcription of inflammatory cytokine genes in M1 macrophages is intriguing. However, in contrast to the rich history of research on the transcriptional activation by Nrf2, the transcriptional inhibition by Nrf2 in proinflammatory macrophages is an emerging concept that are introduced in this study, so that elucidation of fine mechanisms for the Nrf2-mediated inhibition of transcription needs further studies. In this regard, it should be noted that results from luciferase reporter assay imply that whereas Nrf2 acts as a transcriptional activator at various loci, Nrf2 specifically inhibits the inflammation-induced transcription mediated by NF-κB. Of note, our results suggest that Nrf2 interferes with the transcription-activating system optimized for inflammatory response. This notion coincides with the fact that Nrf2 binds to the *IL6* and *IL1b* loci, but does not affect the binding of NF-κB and C/EBPβ to the loci, in our ChIP-seq and ChIP-qPCR analyses.

The binding machinery of Nrf2 to the *IL6* and *IL1b* loci is an important part in the mechanism of Nrf2-mediated transcriptional inhibition. In contrast to the situation that Nrf2 directly binds to a number of detoxifying and antioxidant target genes through ARE and activates expression of these genes, *IL6* gene appears to be regulated in an ARE-independent manner, and AREs near the *IL1b* gene are phylogenetically not conserved. In contrast, our results demonstrate that Nrf2 inhibits the NF-κB-mediated transcription of these proinflammatory cytokine genes. Since the Nrf2-binding regions near the *IL6* and *IL1b* genes correspond to the common binding regions of multiple proinflammatory transcription factors, such as p65, C/EBPβ and c-Jun, it seems likely that Nrf2 is recruited to this region through association with other factors. Although direct interaction of Nrf2 with these inflammatory transcription factors has not been reported, MafK, one of the obligatory partner molecules of Nrf2, is reported to associate with p65 (ref. 36). Consistent with the report, we observed that p65 resides in the Nrf2-based transcription factor complex in LPS-treated Raw264.7 cells (H.S. and H.M., unpublished observation).

Our data further demonstrate that Nrf2 associates with co-activators, such as CBP and the component of Mediator complex, MED16 and MED24, even in the LPS-treated conditions^32^,^33^. Thus, available lines of evidence suggest that in an inflammatory condition, Nrf2 is recruited to regulatory regions of proinflammatory cytokine genes in an ARE-independent manner and inhibits their transcription by interfering with the activity of proinflammatory transcription factors without losing CBP and mediators. Alternative possibility is that Nrf2 suppresses the transcription of proinflammatory cytokine genes indirectly by recruiting transcriptional repressors acting in trans to the transcription factor complex formed in the proximity of the inflammatory cytokine genes.

We surmise that our current findings may account for the mechanisms in drug efficacy of the Nrf2 inducer Tecfidera, which is used for the treatment of multiple sclerosis^12^,^13^, because IL-6 and IL-1 are the key players in the development of multiple sclerosis and other autoimmune diseases. Loss-of-function model mice of IL-6 or IL-1 receptor are resistant to EAE, a murine model of multiple sclerosis^35^,^37^. It has been demonstrated that Nrf2 activation by treatment with small molecule inducers, CDDO (2-cyano-3,12-dioxooleana-1,9(11)-dien-28-oic acid) derivatives and sulforaphane alleviates the EAE clinical score, while *Nrf2*-deficiency leads to increased susceptibility to EAE^38^,^39^,^40^. CDDO-treated EAE mice showed a significant reduction in Th1 and Th17 cytokines including IL-6 (ref. 38), implying that Nrf2 activation with small molecules alleviates EAE through the inhibition of *IL6* gene expression. Our results strongly support the contention that the therapeutic effect of the Nrf2 inducer depends, at least in part, on the Nrf2-mediated inhibition of IL-6 and IL-1 induction. Of note, *Nrf2*-deficiency has been found to cause spontaneous autoimmune phenotypes in certain strains of mice^9^,^10^, raising the possibility that Nrf2 inducers are applicable for the other autoimmune diseases.

We surmise that some of the Nrf2 functions previously attributed to ROS elimination may depend on the repression of IL-6 and IL-1. One of the candidates for this is anti-tumour immunity. It has been shown that Nrf2 acts as a ‘double-edged sword' in tumour development. Nrf2 inhibits carcinogenesis through ROS elimination and detoxification^41^. In contrast, Nrf2 stimulates tumour proliferation through anti-oxidation and metabolic reprogramming of the cells^42^. In this regard, we recently reported that Nrf2 supports anti-tumour immunity by reducing the ROS level in myeloid-derived suppressor cells (MDSCs), resulting in reduced tumour metastasis^43^,^44^. Interestingly, a blockade of IL-6 secreted from MDSCs has been reported to support the anti-tumour immunity^45^. Thus, Nrf2 contributes to the anti-tumour immunity through dual pathways, the repression of IL-6/IL-1 and/or ROS elimination in MDSCs.

Another candidate for the Nrf2 function downstream of the IL-6/IL-1 repression is diabetes prevention. We have reported that genetic Nrf2 activation by *Keap1*-knockdown suppress obesity-induced diabetes through the improvement of insulin secretion and insulin resistance, resulting in the prevention of hyperglycemia^46^. Of note, the blockade of an IL-1 signal with an IL-1 receptor antagonist anakinra (Kineret) improves glycaemia and insulin secretion in patients with type 2 diabetes^47^. Nrf2 activation provokes the alteration of diabetes-related gene expression, which includes anti-oxidation, energy consumption and glyconeogenesis^46^. It seems plausible that in addition to these genes, Nrf2-mediated inhibition of IL-6/IL-1 induction contributes to the prevention and improvement of diabetes.

As IL-6/IL-1 signalling exacerbates a broad spectrum of diseases, an Nrf2 inducer could be beneficial in the treatment of these IL-6/IL-1-related diseases. Neutralizing antibodies and receptor antagonists for blocking IL-6/IL-1 signalling have been developed and approved, for instance, for rheumatoid arthritis and other inflammatory diseases. Efforts are underway to find new indications, such as diabetes and chronic obstructive pulmonary disease^1^,^2^,^48^. Whereas these antibody-based drugs need to be administered subcutaneously or intravenously, one of the advantages of the small molecule Nrf2 inducers is that they can be administered orally, lowering the burden on patients. Therefore, it should be noted that our present study has firmly established the concept that Nrf2 inducers retain the potential to act as alternative anti-inflammatory agents.

## Methods

### Experimental animals

Deletion of the floxed *Keap1* gene in myeloid lineage cells was achieved by crossing *Keap1* flox mice^20^ with heterozygous mice harbouring a *Cre* recombinase transgene under the regulation of the *lysozyme M* (*LysM*) locus^21^. WT, *Nrf2*^*−/−*^ (Nrf2-KO) (ref. 3) and *Keap1*^*flox/flox*^::*LysM*-Cre (KpCKO) mice were maintained in a C57BL6/J genetic background. WIM-6 (ref. 34), Nrf2-KO and *Keap1*-knockdown mice (*Keap1*^flox/flox^)^49^ were maintained in C57BL6/J and ICR background. Results were similar in both sexes. Mice were used for experiments at 8–15 weeks of age, with the exception of neutrophil count that was performed with mice <1 year old. All mice were treated according to the regulations of The Standards for Human Care and Use of Laboratory Animals of Tohoku University (Sendai, Japan) and the Guidelines for Proper Conduct of Animal Experiments of the Ministry of Education, Culture, Sports, Science and Technology of Japan. All animal experiments were executed with the approval of the Tohoku University Animal Care Committee.

### BMDMs and cell cultures

To obtain BMDMs, hemolyzed bone marrow cells from WT, *Nrf2*^*−/−*^ (Nrf2-KO) and *Keap1*^*flox/flox*^::*LysM*-Cre (KpCKO) mice were cultured for 7 days in DMEM supplemented with 10% (vol/vol) FBS (fetal bovine serum), 20-ng ml^−1^ M-CSF (macrophage colony-stimulation factor, PeproTech) and Antibiotic-Antimycotic (Life Technologies). At day 3, a half volume of fresh DMEM with 10% (vol/vol) FBS and 20-ng ml^−1^ M-CSF was added. At day 7, BMDMs were depleted with M-CSF several hours before stimulation. Unless stated, data are presented as the mean±s.d. of three mice, and the significance was determined by Student's unpaired *t*-test. THP-1 cells were obtained from the Japanese Cancer Research Resources Bank, and maintained in RPMI 1640 containing 10% (vol/vol) FBS. Data from THP-1 cells are presented as the mean±s.d. of three individual samplings, and the significance was determined by Student's unpaired *t*-test.

### Reagents and stimulation

Cells were left untreated (Con) or incubated with 5-ng ml^−1^ LPS and 10-ng ml^−1^ IFNγ (M1), 10-ng ml^−1^ IL-4 (M2), or 5-ng ml^−1^ LPS (LPS). LPS from *Escherichia coli* 0111:B4 was purchased from Sigma-Aldrich, and IFNγ and IL-4 were from R&D Systems. For pharmacological Nrf2 activation, cells were treated with 100-μM diethylmaleate (DEM) or 15-deoxy-Δ^12,14^-prostaglandin *J*_2_ (15d-PGJ_2_). These Nrf2 inducers were stored as the solutions dissolved in DMSO (vehicle). DEM was from Wako chemicals, and 15d-PGJ_2_ was from Sigma-Aldrich. For detection of ROS, BMDMs were treated with CellROX Deep red reagent (Thermo) during the last 30 min of 4 h stimulation with or without 5-ng ml^−1^ LPS concomitant treatment with 100-μM DEM or 1-mM NAC. CellROX signal was detected with flow cytometry analysis using FACS-Caliber (BD Biosciences) and FlowJo software (TOMY Digital Biology). For the evaluation of the mRNA stability, 5-μg ml^−1^ actinomycin D (Sigma-Aldrich) was added to cell cultures pre-treated with LPS for 3 h without removal of the LPS-containing media. Unless stated, cells were stimulated for 6 h in microarray and qRT-PCR analyses, and for 4 h in ChIP-seq, ChIP-qPCR and immunoblot analyses.

### Immunoblot analysis

Whole cells were lysed in a SDS-containing sampling buffer and subjected to immunoblot analysis using Nrf2-specific antibody^50^ (clone 103, 1:200 dilution) and α-Tubulin-specific (Sigma-Aldrich; T9026, 1:5,000 dilution) antibodies. Immunoblot using α-Tubulin-specific antibody is used as a loading control. Images have been cropped for presentation. Full-size images are presented in Supplementary Fig. 11.

### Microarray analysis

Total RNA samples of BMDMs from two independent mice of each genotype were extracted using an RNeasy Micro Kit (Qiagen) and used for the microarray analyses. The expression data were normalized using GeneSpring software (Silicon Genetics), and analysed using an R/bioconductor (ver. 3.0.2, R Core Team (2014). R: A language and environment for statistical computing. R Foundation for Statistical Computing, Vienna, Austria. URL http://www.R-project.org/). Pathway analysis was performed using the Kyoto Encyclopedia of Genes and Genomes^26^.

### Real-time quantitative PCR analysis

Total RNA was isolated from cells using ISOGEN (Nippon Gene). First-strand cDNA was synthesized from total RNA using SuperScript III Reverse Transcriptase (Life Technologies). Real-time quantitative PCR was performed with an ABI PRISM 7300 sequence detector system and StepOne Plus (Applied Biosystems) using THUNDERBIRD SYBR qPCR Mix (TOYOBO). Data were normalized by the *Gapdh* mRNA level. Primer sequences are available on request.

### Chromatin immunoprecipitation and ChIP-sequencing (ChIP-seq)

BMDMs were fixed with 1% (wt/vol) formaldehyde and subsequently quenched with glycine. After washing with PBS, the fixed samples were suspended in cell lysis buffer (5-mM PIPES-KCl pH8.0, 85-mM KCl, 0.5% (vol/vol) NP40), and collected nuclei were stored at −80 °C as nuclear pellets or nuclear lysates dissolved in nuclei lysis buffer (50-mM Tris-HCl pH8.0, 10-mM EDTA, 1% (wt/vol) SDS). For Nrf2 ChIP, lysates were thawed and sonicated with Sonifier (BRANSON) to obtain chromatin fragments of 300–1,000 bp, and 10-fold-diluted with ChIP dilution buffer (16.7-mM Tris-HCl pH8.0, 1.2-mM EDTA, 0.01% (wt/vol) SDS, 1.1% (vol/vol) TrironX100, 167-mM NaCl). For Pol II, p65, C/EBPβ, H3K27me3, H3K27Ac and H3K4me3 ChIP, nuclear pellets were thawed and suspended in buffer NUC (15-mM Hepes pH 7.5, 60-mM KCl, 15-mM NaCl, 0.32-mM sucrose, 3-μM CaCl_2_) and digested with MNase (New England Biolabs). After doubling-dilution with a sonication buffer (90-mM Hepes pH 7.9, 220-mM NaCl, 10-mM EDTA, 1% (vol/vol) NP-40, 0.2% (wt/vol) sodium deoxycholate, 0.2% (wt/vol) SDS), nuclei were homogenized with a Bioruptor (Tosho denki). After washing, a complex of nuclear proteins/DNAs and antibodies against Nrf2 (Cell Signaling technology; D1Z9C; 1:250 dilution), Pol II (Santa Cruz; sc-9001; 1:24 dilution), p65 (Cell Signaling technology; D14E12; 1:120 dilution), C/EBPβ (Santa Cruz; sc-150; 1:24 dilution), H3K27me3 (Cosmo Bio; MABI0323; 1:240 dilution), H3K27Ac (Cosmo Bio; MABI0309; 1:240 dilution) and H3K4me3 (Cosmo Bio; MABI0004; 1:240 dilutions) was retrieved with Protein A- and Protein G-Dynabeads (Life Technologies). After the cross-linking was reversed, chromatin fragments were treated with RNase A and proteinase K. DNA was purified with phenol-chloroform extraction or Ampure XP (Beckman Coulter). In the ChIP-qPCR analyses, the values from the immunoprecipitated samples were normalized to that from the input DNA. Primer sequences are available on request. ChIP-seq libraries were prepared from ∼1 ng each of ChIP and input samples using the Ovation Ultralow DR Multiplex System 1–8 (NuGEN, 0330-32). Libraries were clonally amplified in a flow cell and sequenced with Illumina Hiseq 2500 (Illumina) to generate paired-end sequences. BMDMs from two independent mice were used for the ChIP-seq, and the sum of sequenced reads from two samples was subjected to following analyses. Sequenced reads were mapped to the mouse genome^51^ (mm9) with bwa^52^ (ver. 0.6.2-r126). Paired reads uniquely mapped to the genome were extracted using samtools^53^ (ver. 0.1.18). The peaks were called using a model-based analysis of ChIP-seq (macs^54^, ver. 2.1.0) using *P*<0.0005 as a threshold. The Nrf2 ChIP-seq data of Hepa1 cells obtained in a previous study were re-peak called in the same manner. Peaks were annotated with BEDtools^55^ and a ChIPpeakAnno package^56^ (ver. 2.10.0). For the *de novo* motif discovery, the sequences of the −25-bp to +25-bp region from peak summits were analysed with a rGADEM package (ver. 2.0.10, Arnaud Droit, Raphael Gottardo, Gordon Robertson and Leiping Li (2014). rGADEM: *de novo* motif discovery).

### ELISA

BMDMs were incubated with 5-ng ml^−1^ LPS and 100-μg ml^−1^ alum (Thermo Scientific; Imject Alum Adjuvant) with or without DEM for 24 h. The concentration of IL-6 and IL-1β in the culture medium was measured using an ELISA kit (R&D Systems), according to the manufacturer's instructions.

### Luciferase reporter assay

Approximately 1 kb upstream region of the *IL6* gene TSS containing the WT ARE (GCTGAGTCA) or mutated ARE (AGATCTGAC) was conjugated to the translation start site of the NanoLuc gene in the pNL2.2 vector (Promega). 293T cells (2.0 × 10^3^ cells per well) were plated in 96-well plates 24 h before transfection. The NanoLuc reporter vectors were co-transfected with pQC-FLAG-hNRF2^T80R^ plasmid^33^ and pcDNA3-p65 plasmid using Lipofectamin 3000 Reagent (Thermo) according to the manufacturer's protocol. After 48 h of the transfection, the luminescence was quantified and normalized using Nano-Glo Dual-Luciferase Reporter Assay (Promega).

### Immunoprecipitation of Nrf2 complex in Raw264.7 cells

Raw264.7 cells were cultured in RPMI1640 supplemented with 10% FBS. Right before the treatment with DEM and LPS, the medium was changed to low-glucose-DMEM supplemented with 10% FBS. An amount of 100-μM of DEM with or without 20-ng ml^−1^ LPS were added to the medium, and the cells were harvested after 4 h. The cells were washed with PBS and crosslinked with 0.5-mM DTME and 0.5-mM DSP, which were quenched by 5-mM cysteine after 30 min. The crosslinked cells were lysed in RIPA buffer, and the supernatant was collected and used for immunoprecipitation with 5-μg antibody specific for Nrf2 (Cell Signaling Technology; D1Z9C-XP) per sample. Precipitated samples together with input samples were analysed by immunoblot analysis, for which anti-Nrf2 (ref. 50) (1:100), anti-CBP (Santa Cruz; sc-1211, 1:2,000), anti-MED16 (Abcam; ab130996, 1:5,000) and anti-MED24 (Bethyl lab; A301-472A, 1:2,000) antibodies were used.

### Deletion of Nrf2-binding regions with CRISPR/Cas9 system

The expression vector to express the guide RNA and Cas9 was produced using px330 plasmid. Raw264.7 cells were co-transduced with two types of px330 (ref. 57) those express guide RNAs specific for upstream and downstream regions of Nrf2-binding region, and selected with puromycin.

### *In vivo* inflammatory models and *IL6*-luc imaging analyses

Mice were subcutaneously administered with 150-μg myelin oligodendrocyte glycoprotein 35–55 peptide (AnaSpec) and complete Freund's adjuvant (Difco) containing 0.8-mg *Mycobacterium tuberculosis* in the abdomen. To support penetration of the blood–brain barrier, 200-ng pertussis toxin was intraperitoneally administered on days 0 and 2. EAE clinical signs were scored as follows: 0, normal; 1, limp tail or hind limb weakness; 2, both of limp tail and hind limb weakness; 3, partial hind limb paralysis; and 4, complete hind limb paralysis. *In vivo IL6*-luc luminescence was measured with an IVIS SpectrumCT (PerkinElmer) after intraperitoneal injection of luciferin (Promega). For quantification, *IL6*-luc luminescence from the ventral midline area of EAE-induced mice was measured with Living Image version 4.3. Mice on the C57BL/6 background were shaved on their back at the day before imaging analysis. For the cutaneous *S. aureus* infection model, mice on the ICR background were shaved on their back and inoculated intra-dermally with mid-logarithmic growth phase *S. aureus* 834 strain (3 × 10^6^ colony forming units) in 100-μl of sterile PBS using a 27-gauge syringe. *In vivo* bioluminescence was evaluated at 0, 2, 24 and 48 h after inoculation.

### Data availability

ChIP-seq data are deposited to DDBJ (DNA Data Bank of Japan) under accession number DRA003771, and microarray data are deposited to GEO (Gene Expression Omnibus) under accession number GSE71263. The data that support other findings of this study are available from the corresponding author upon request.

## Additional information

**How to cite this article:** Kobayashi, E. H. *et al*. Nrf2 suppresses macrophage inflammatory response by blocking proinflammatory cytokine transcription. *Nat. Commun.* 7:11624 doi: 10.1038/ncomms11624 (2016).

## Supplementary Material

Supplementary Information Supplementary Figures 1 - 11 and Supplementary References 1 - 6

Peer Review File
