# Supplementary material for: Nrf2 suppresses macrophage inflammatory response by blocking proinflammatory cytokine transcription
Source: Nat Commun. 2016 May 23;7:11624. doi: 10.1038/ncomms11624 (PMC4879264; doi:10.1038/ncomms11624)
Supplement: Supplementary Information — Supplementary Figures 1 - 11 and Supplementary References 1 - 6 [file ncomms11624-s1.pdf]

Supplementary Fig. 1

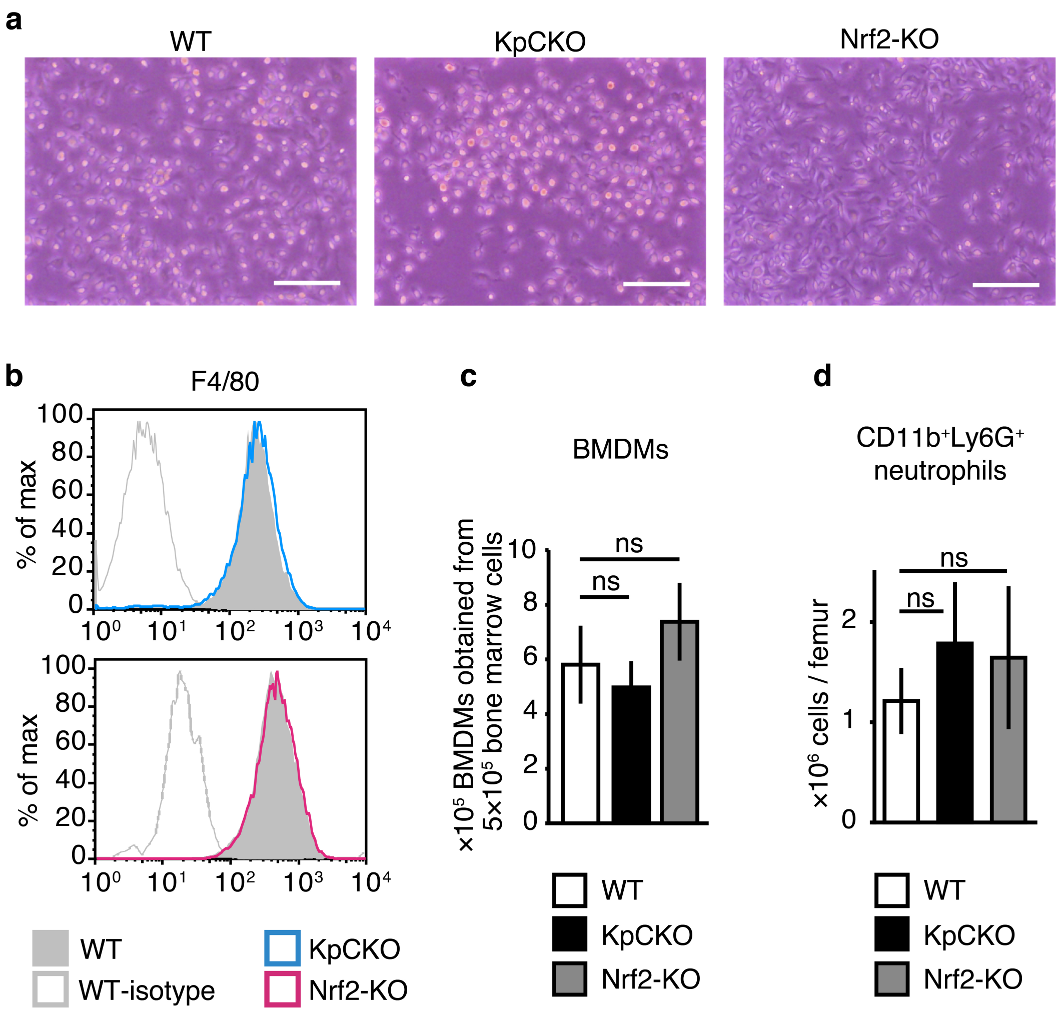

Supplementary Fig. 1 *Nrf2*-deficient and *Keap1*-deficient BMDMs are normal in appearance, cell numbers and macrophage marker F4/80 expression. (**a**) Images of BMDMs obtained from wild-type (WT), KpCKO and Nrf2-KO mice. Scale bars represent 100 μm. (**b**) Flow cytometric analysis to confirm the expression of macrophage marker F4/80. Note that BMDMs from all genotypes of mice are almost 100% positive for F4/80. Fluorescein isothiocyanate- or phycoerythrin-conjugated F4/80-specific monoclonal antibodies were purchased from eBioscience. Flow cytometry analysis was performed using FACS-Caliber (BD Biosciences) and FlowJo software (TOMY Digital Biology). (**c**) The numbers of BMDMs obtained from 5×10^5 bone marrow cells of mice with each genotype. BMDMs were non-enzymatically

dissociated from dishes and counted with Countess (Thermo). Almost all of these BMDMs are F4/80-positive as shown in (**b**). (**d**) The numbers of neutrophils obtained from unilateral femur of mice with each genotype. Bone marrow mononuclear cells obtained with Histopaque (Sigma-Aldrich) were counted and used for flow cytometry analysis. The number of neutrophil was calculated from the total cell count and the percentage of $CD11b^{+}Ly6G^{+}$ cells. Representative data (**a, b**) or mean±s.d. (**c**) from at least four mice for macrophage count and mean±s.d. from 3-4 mice (**d**) for neutrophil count are shown (ns, not significant by unpaired *t*-test).

Supplementary Fig. 2

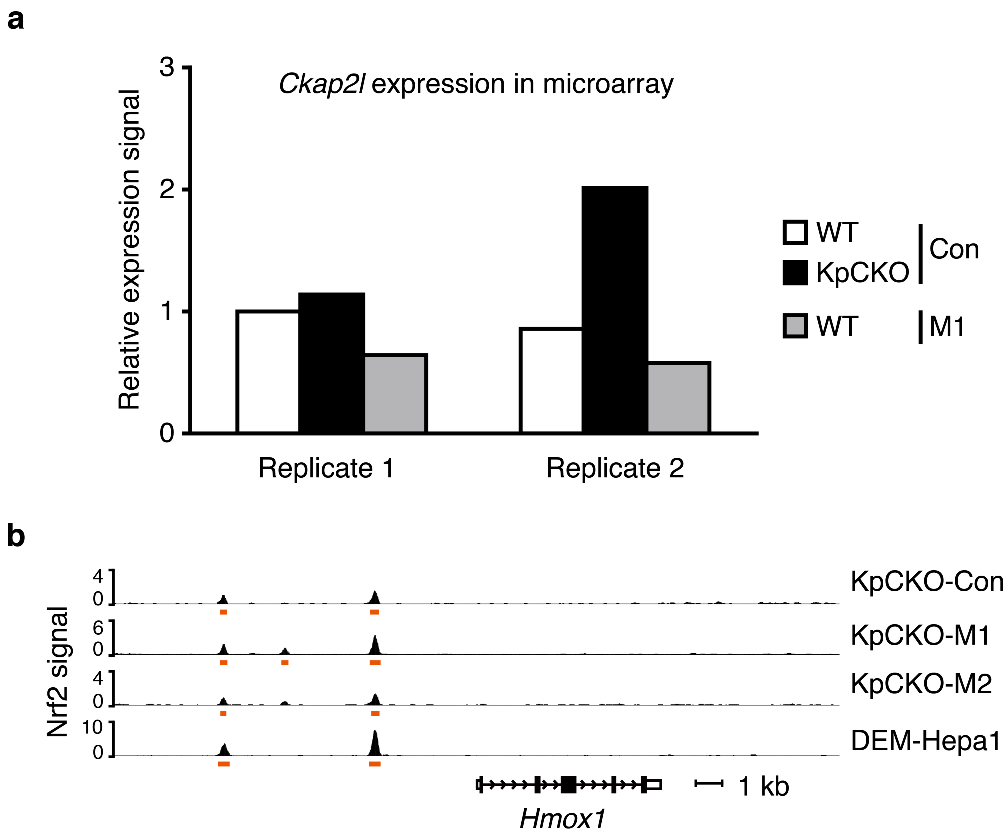

Supplementary Fig. 2 Microarray and ChIP-seq analyses of *Keap1*-deficient BMDMs. (**a**) Relative expression signal of *Ckap2l* gene in microarray analysis. Wild-type (WT) and *Keap1*-deficient BMDMs (KpCKO) were untreated (Con) or treated with M1 stimulation (M1, 5-ng ml^{-1} LPS and 10-ng ml^{-1} IFNγ) for 6 hours. (**b**) Nrf2 ChIP-seq track of known Nrf2 target gene *Hmox1*. *Keap1*-deficient BMDMs were untreated (KpCKO-Con) or treated with M1 stimulation (KpCKO-M1, 5-ng ml^{-1} LPS and 10-ng ml^{-1} IFNγ) or M2 stimulation (KpCKO-M2, 10-ng ml^{-1} IL-4) for 4 hours. ChIP-seq data of DEM-treated Hepa1 cells were reported elsewhere and re-analysed[1]. Orange bars under the tracks represent the peak regions detected by the MACS2 peakcaller.

Supplementary Fig. 3

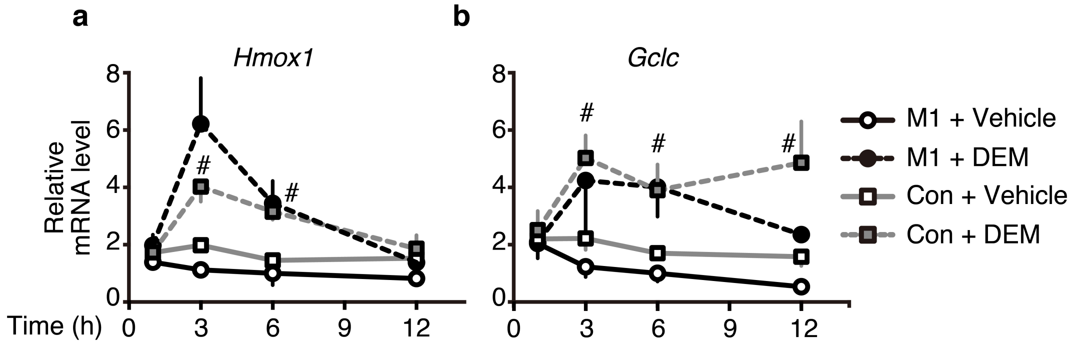

Supplementary Fig. 3 Time-course expression of known Nrf2 target genes. (**a-b**) Relative mRNA expression of antioxidative Nrf2 target genes, *Hmox1* (**a**) and *Gclc* (**b**), at indicated times from the start of M1 stimulation (M1, 5-ng ml^{-1} LPS and 10-ng ml^{-1} IFNγ) in the presence of 100-μM DEM or vehicle. DEM and vehicle were added in the culture media at the same time as M1 stimulation. The value of M1+Vehicle at 6 hours is set to 1. **P*<0.05 (unpaired *t*-test) against M1+Vehicle, #*P*<0.05 (unpaired *t*-test) against Con+Vehicle. Data are mean±s.d. from three mice.

Supplementary Fig. 4

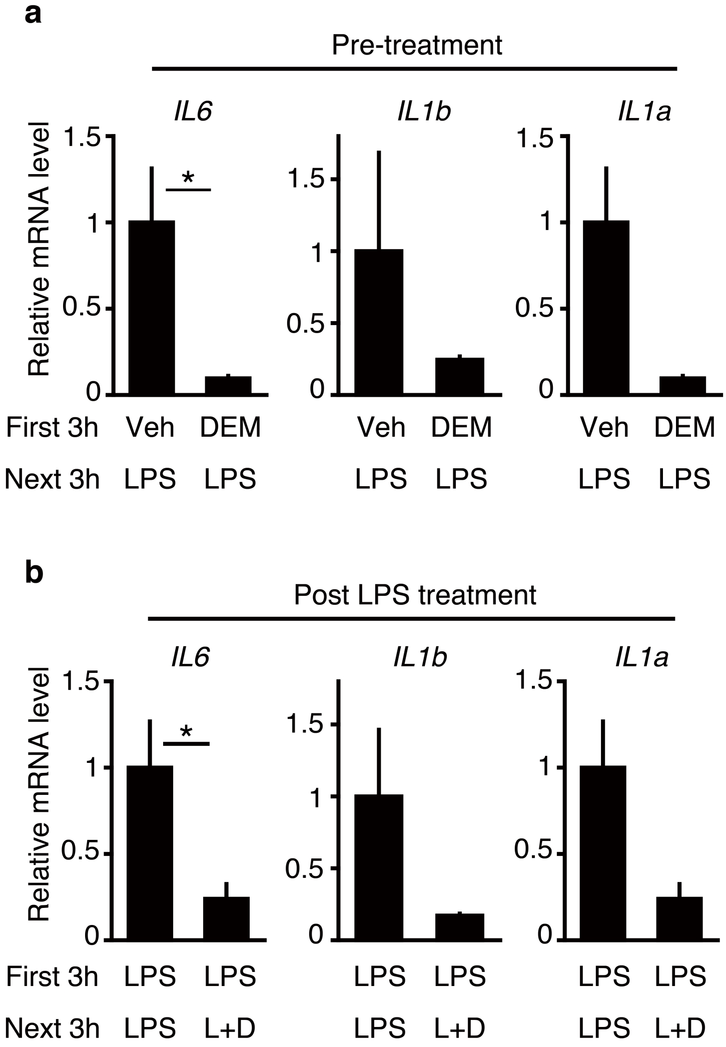

Supplementary Fig. 4 Nrf2 inhibits expression of proinflammatory cytokine genes regardless of the order of Nrf2 activation and LPS treatment. (**a**) Relative mRNA expression of the *IL6*, *IL1b* and *IL1a* genes in the wild-type BMDMs pretreated with 100-μM DEM (DEM) or vehicle (Veh) for 3 hours before 3 hours of 5-ng ml^{-1} LPS treatment. (**b**) Relative mRNA expression of the *IL6*, *IL1b* and *IL1a* genes in the wild-type BMDMs treated with DEM or vehicle in combination with LPS for 3 hours after 3 hours of LPS treatment. L+D, LPS+DEM. Data are mean±s.d. from three mice (**P*<0.05, unpaired *t*-test). This repression was reproducible in BMDMs obtained from multiple mice.

Supplementary Fig. 5

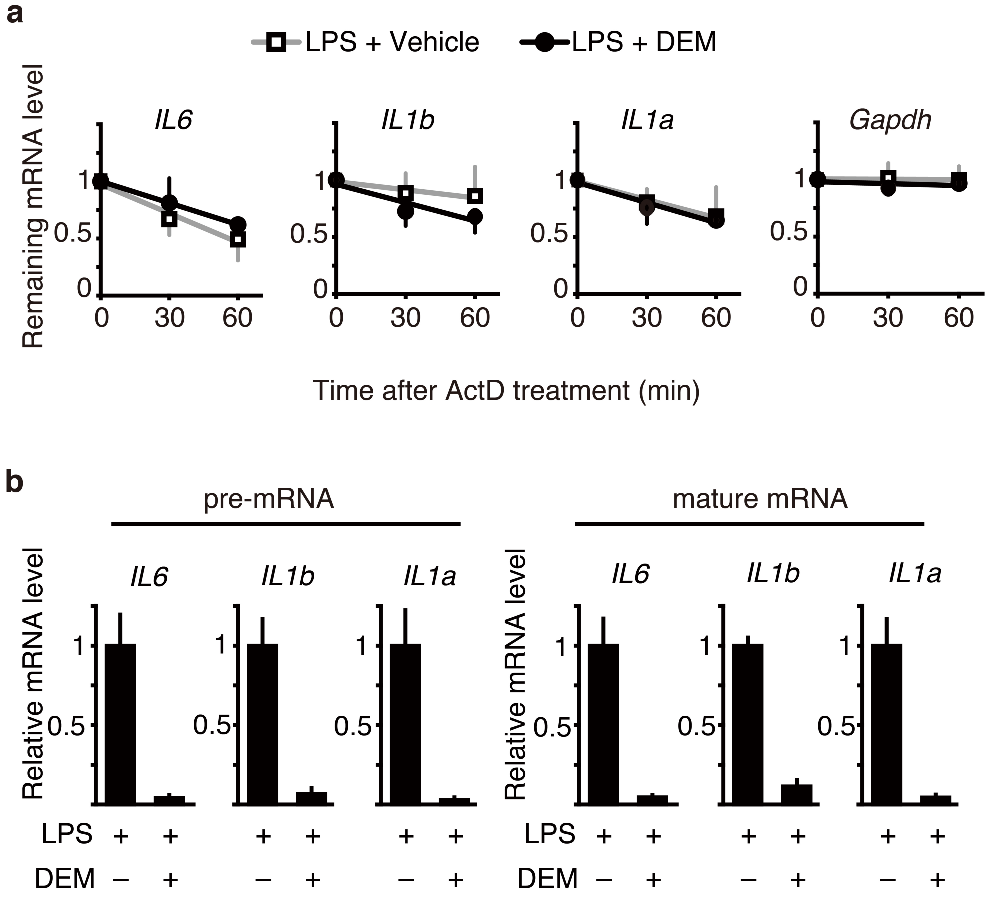

Supplementary Fig. 5 Nrf2 inhibits the expression of proinflammatory cytokines at the transcriptional level. (**a**) Stability of mRNA was not changed by the chemical Nrf2 inducer. Wild-type BMDMs were LPS-stimulated (5-ng ml^{-1}) for 3 hours in the presence of 100-μM DEM before the treatment with actinomycin D (ActD). The stability of mRNA is evaluated by the remaining mRNA after ActD treatment. Data are normalized by the mRNA level of each sample at 0 min. The remaining mRNA level of *Gapdh* is used as a control of stable mRNA. (**b**) Reduction in pre-mRNA of proinflammatory cytokine genes by the chemical Nrf2 inducer. Wild-type BMDMs were LPS-stimulated in the presence of 100-μM DEM or vehicle for 6 hours. Pre-mRNA expression was detected by RT-qPCR using the primers specific to the introns of *IL6*, *IL1b* and *IL1a* genes. Data are mean±s.d. from three mice (**P*<0.05, unpaired *t*-test).

Supplementary Fig. 6

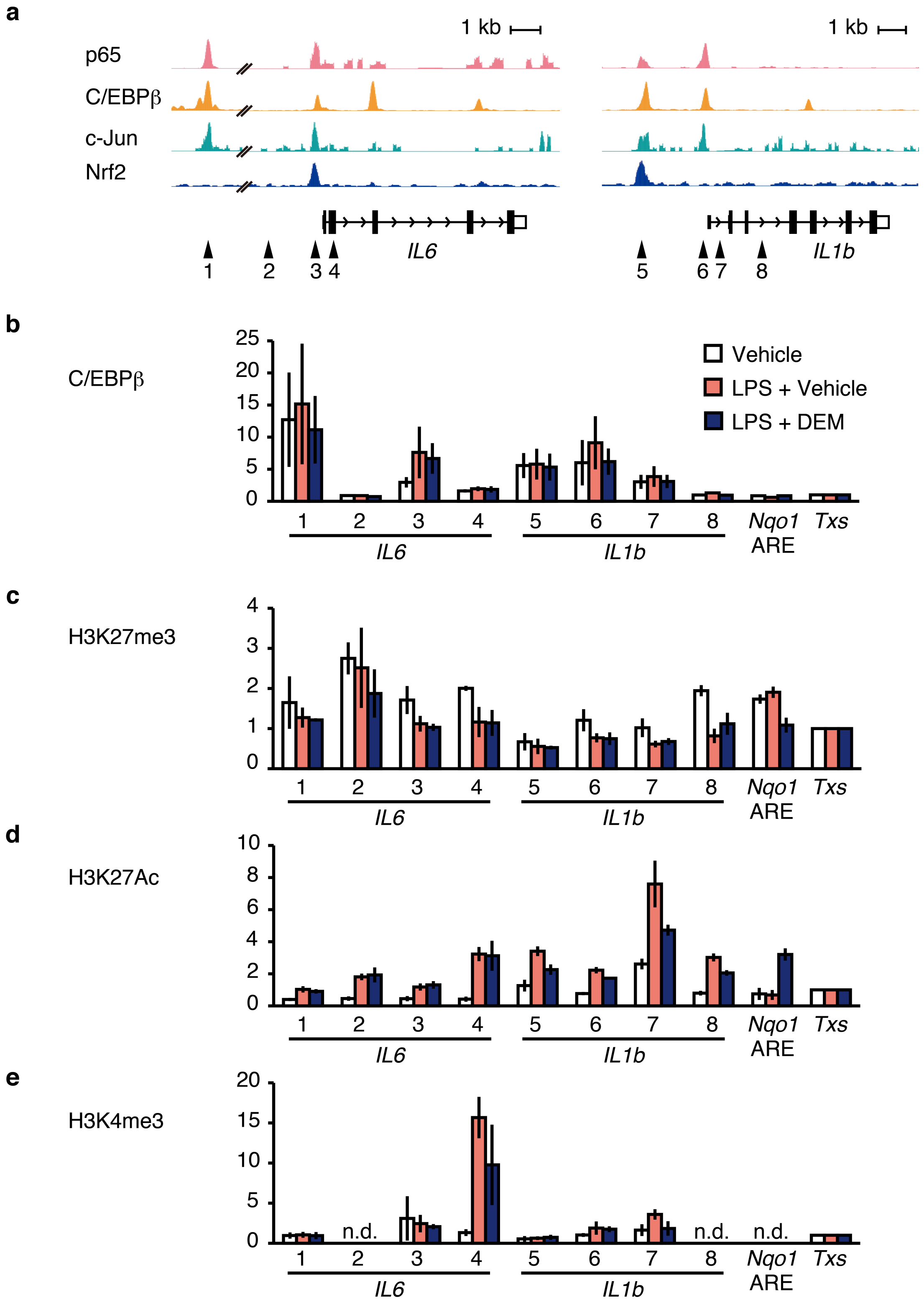

Supplementary Fig. 6 Nrf2 activation exerts negligible effect on the histone modifications and recruitment of C/EBPβ at the *IL6* and *IL1b* loci. (**a**) ChIP-sequencing track specific for p65[2], C/EBPβ[3] and c-Jun[4] at the *IL6* and *IL1b* loci. Nrf2 ChIP-sequencing track of KpCKO-M1 BMDMs is depicted as in **Fig. 2**. (**b-e**) ChIP-qPCR for C/EBPβ (**b**), H3K27me3 (**c**), H3K27Ac (**d**), and H3K4me3 (**e**). Wild-type BMDMs were stimulated with 5-ng ml^{-1} LPS, in the presence of 100-μM DEM or vehicle, for 4 hours. Primers for the ChIP-qPCR were designed to amplify the regions indicated with triangles in (**a**). The *Txs* intron and the Nrf2-binding site near *Nqo1* gene (*Nqo1* ARE) were used as negative controls. The 60-kb upstream site of the *IL6* gene is used as a positive control for C/EBPβ binding. Data are shown as relative enrichment, and the signal at the *Txs* intron is set to 1. n.d., not determined.

Supplementary Fig. 7

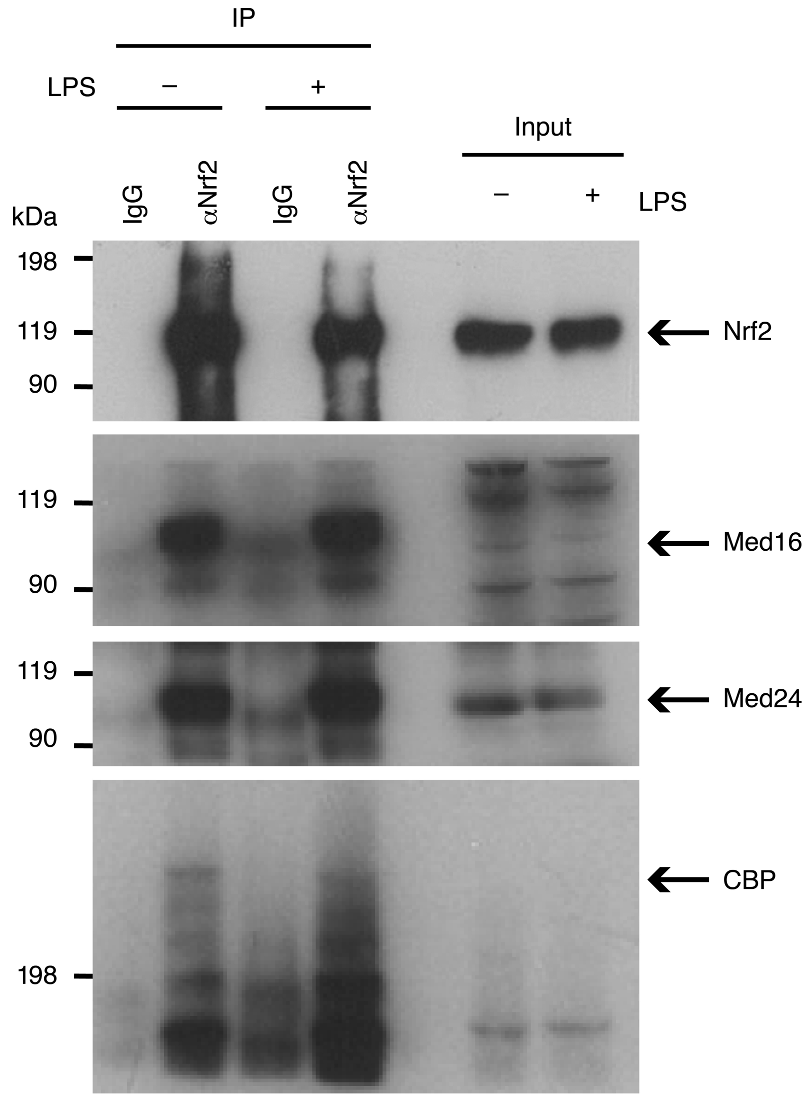

Supplementary Fig. 7 Co-activator CBP and components of Mediator complex, MED16 and MED24, reside in immunoprecipitated endogenous NRF2 complex in Raw264.7 cells in the presence of DEM. CBP[5] and the component of Mediator complex[6], MED16 and MED24, have been shown to associate with Nrf2 and act as transcriptional co-activators. We immunoprecipitated endogenous NRF2 complex in Raw264.7 cells treated with 100-μM DEM and with or without 20-ng ml^{-1} LPS for 4 hours. Note that MED16/24 and CBP in the Nrf2 complex are rather increased upon treatment with LPS.

Supplementary Fig. 8

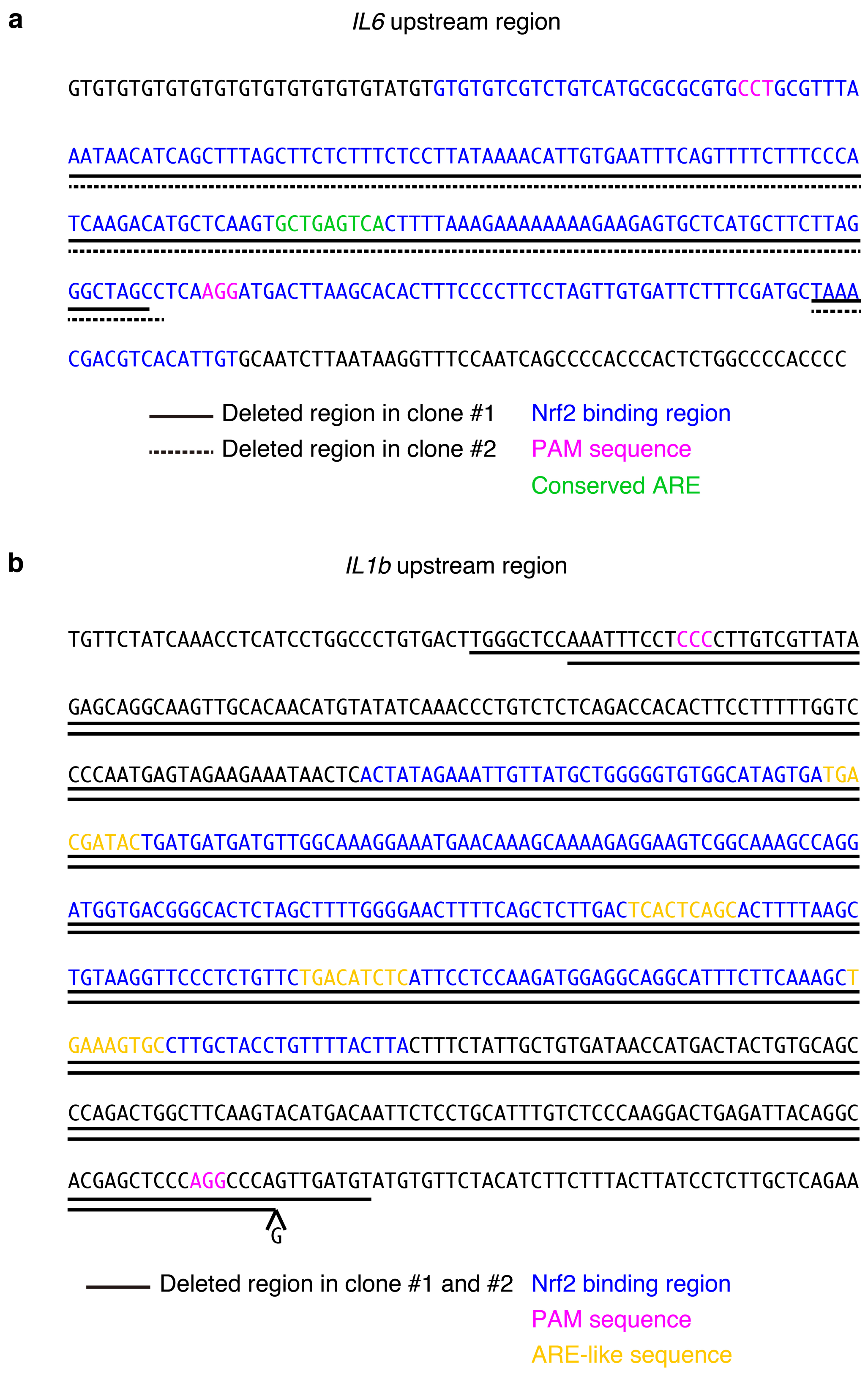

Supplementary Fig. 8 Deletions of regulatory sequences of *IL6* and *IL1b* genes harboring AREs from Raw264.7 cells. Regulatory sequences harboring AREs of the *IL6* (**a**) and *IL1b* (**b**) genes were deleted by means of genome-editing technology. Lines under the sequences show the deleted region. The regulatory regions determined in KpCKO-M1 BMDMs are depicted with blue characters, while PAM sequences used for deletion are depicted with magenta. In the cells lack the *IL6* gene regulatory region (**a**), upper line shows deleted region from clone 1, while lower dashed line shows deleted region in clone 2. Green characters show the conserved ARE, TGAG/CnnnGC. In the cells lack the *IL1b* regulatory region (**b**), both upper and lower lines show deleted regions in clones 1 and 2. Yellow characters show the ARE-candidate sequences.

Supplementary Fig. 9

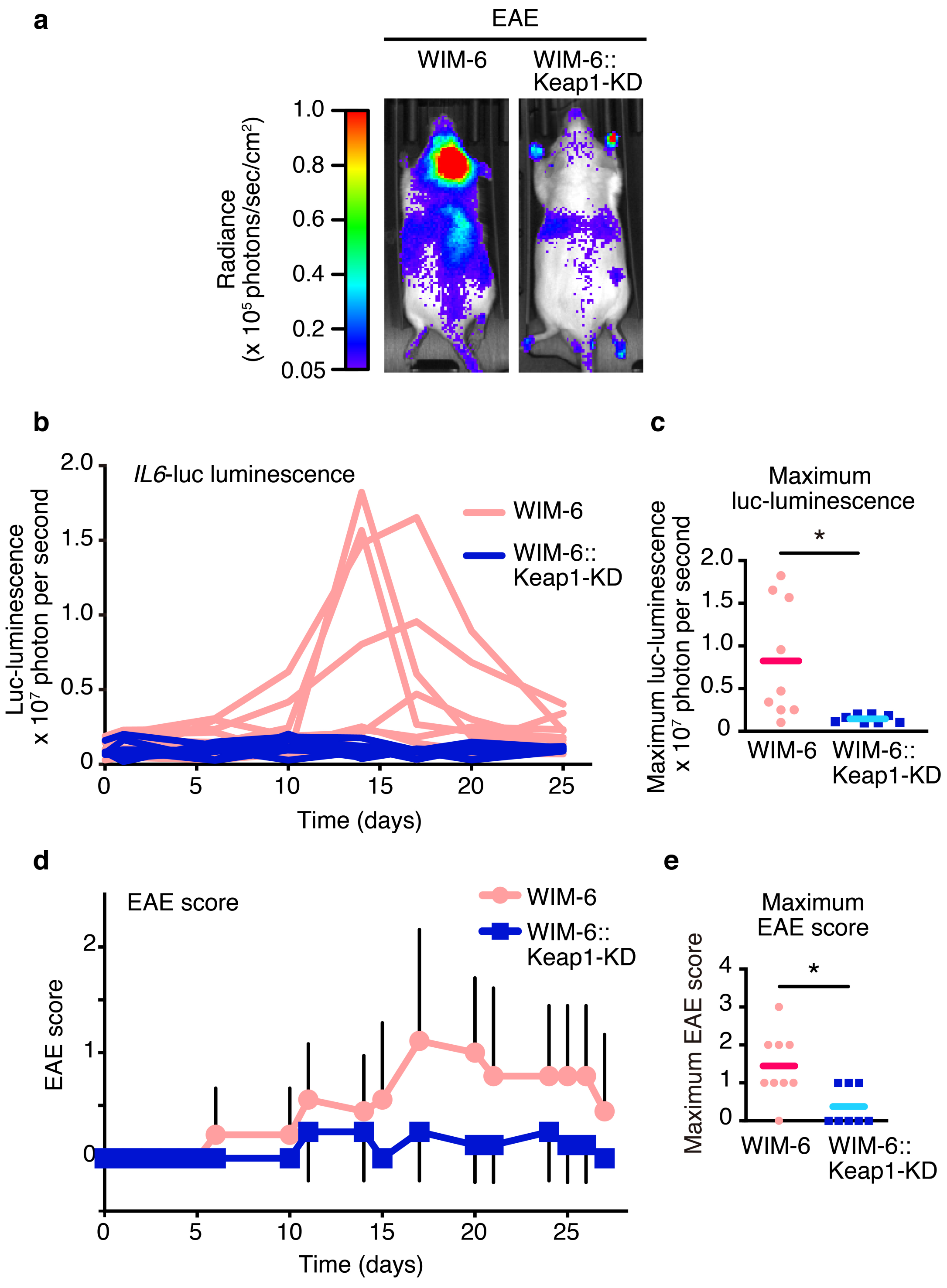

Supplementary Fig. 9 Nrf2 inhibits *IL6* expression and inflammatory phenotypes in EAE model with the ICR background. (**a**-**e**) *In vivo* monitoring of *IL6* expression in the EAE model. WIM-6 mice with wild-type *Keap1* (WIM-6, n=9) or *Keap1*-knockdown background (WIM-6::Keap1-KD, n=8) on the ICR background were subjected to EAE induction. Representative images of *IL6*-luc luminescence at day 17 (**a**) and the *IL6*-luc luminescence intensity in each mouse (**b**), and the maximum *IL6*-luc luminescence in the observation period are shown (**c**). Red and blue lines represent the mean of the maximum luminescence intensity. *$P<0.05$, unpaired *t*-test. (**d, e**) Clinical EAE scores in the same experiment as **a-c**. The mean±s.d. of EAE score (**d**) and the maximum EAE score in the observation period (**e**) are shown. Red and blue lines represent the mean of the maximum EAE score. *$P<0.05$, unpaired *t*-test.

Supplementary Fig. 10

a

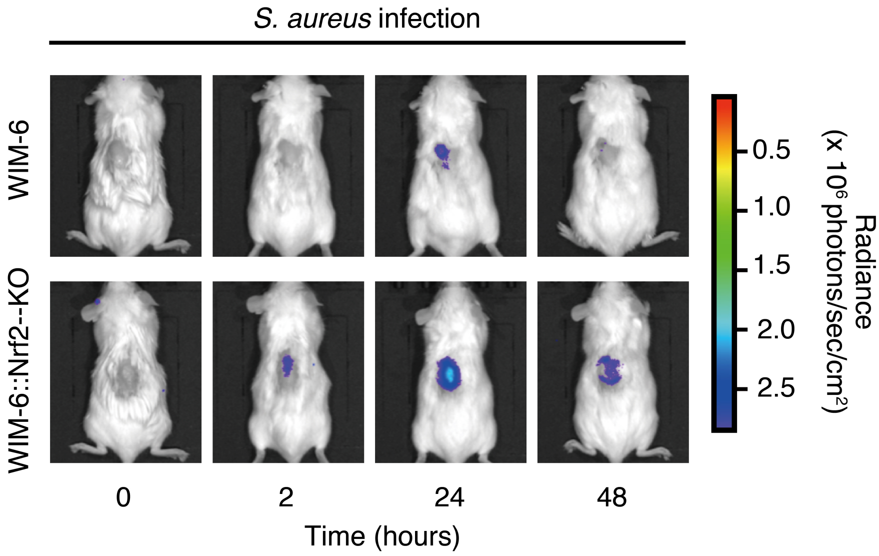

b

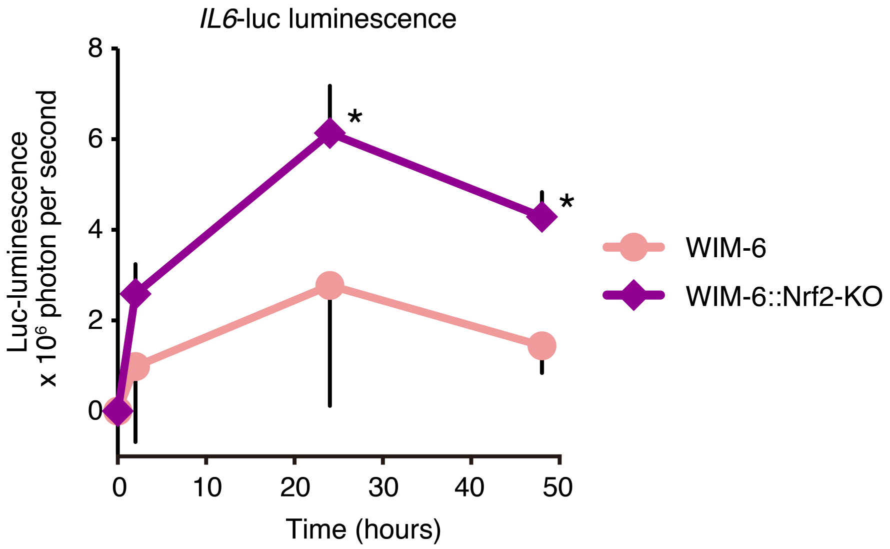

Supplementary Fig. 10 Nrf2 knockout mice show enhanced *IL6* expression *in vivo*. *In vivo* monitoring of *IL6* expression was conducted exploiting a *S. aureus* infection model. WIM-6 mice with wild-type *Nrf2* (WIM-6, n=5) or *Nrf2*-knockout background (WIM-6::Nrf2-KO, n=4) on the ICR background were infected with *S. aureus*. Representative *in vivo* bioluminescence (**a**) and time-course of luminescence intensity (**b**) are shown. Note that the Nrf2 knockout mice show markedly enhanced inflammation, as can be seen in terms of luminescence mimicking the *IL6* gene expression. *P<0.05, unpaired t-test.

Supplementary Fig. 11

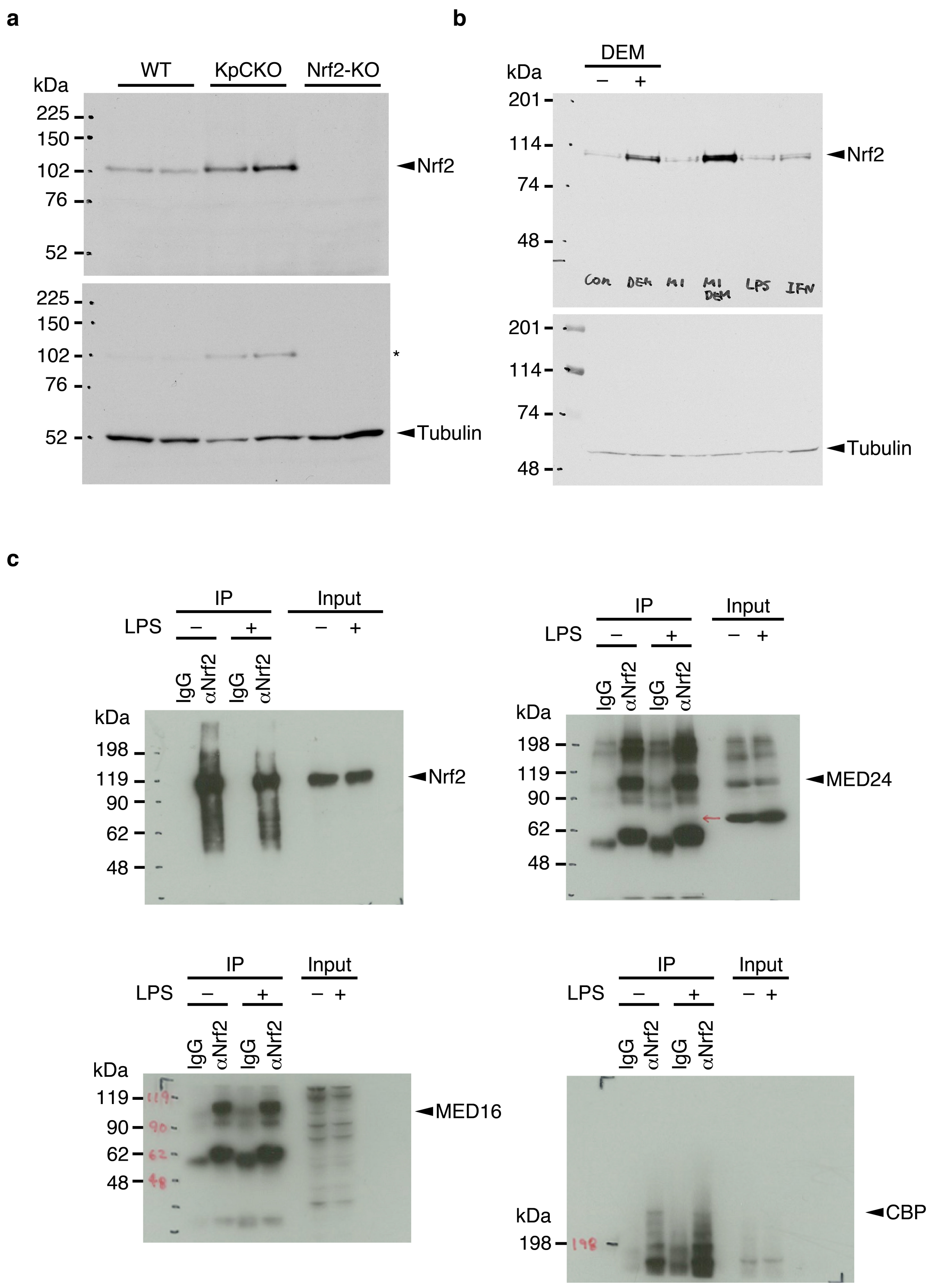

Supplementary Fig. 11 Full-size images of Western blots. (**a**) Uncropped scan of Western blot shown in Fig. 1a. Nrf2 protein expression in BMDMs from two independent wild-type (WT), KpCKO, and Nrf2KO mice, respectively. The left 4 lanes were cropped for Fig. 1a. After first immunoblotting to detect Nrf2, the membrane was incubated with antibody specific for α-Tubulin to confirm equal loading. *Remaining signal of Nrf2 protein. (**b**) Uncropped scan of Western blot shown in Fig. 4a. Nrf2 protein accumulation in the WT BMDMs treated with 100-μM DEM or vehicle for 4 hours. The left 2 lanes were cropped for Fig. 4a. After first immunoblotting to detect Nrf2, the membrane was stripped and incubated with antibody specific for α-Tubulin to confirm equal loading. (**c**) Uncropped scans of Western blots in Supplementary Fig. 7. Endogenous NRF2 complex was immunoprecipitated from DEM-treated Raw264.7 cells treated with or without LPS.

References

1. Hirotsu, Y. *et al.* Nrf2-MafG heterodimers contribute globally to antioxidant and metabolic networks. *Nucleic Acids Res.* **40,** 10228–39 (2012).
2. Barish, G. D. *et al.* Bcl-6 and NF-kappaB cistromes mediate opposing regulation of the innate immune response. *Genes Dev.* **24,** 2760–5 (2010).
3. Roe, J.-S., Mercan, F., Rivera, K., Pappin, D. J. & Vakoc, C. R. BET Bromodomain Inhibition Suppresses the Function of Hematopoietic Transcription Factors in Acute Myeloid Leukemia. *Mol. Cell* **58,** 1028–39 (2015).
4. Uhlenhaut, N. H. *et al.* Insights into negative regulation by the glucocorticoid receptor from genome-wide profiling of inflammatory cistromes. *Mol. Cell* **49,** 158–71 (2013).
5. Katoh, Y. *et al.* Two domains of Nrf2 cooperatively bind CBP, a CREB binding protein, and synergistically activate transcription. *Genes to Cells* **6,** 857–868 (2001).
6. Sekine, H. *et al.* The Mediator Subunit MED16 Transduces NRF2-activating Signals into Antioxidant Gene Expression. *Mol. Cell. Biol.* (2015). doi:10.1128/MCB.00785-15
