# Supplementary material for: Nrf2 suppresses macrophage inflammatory response by blocking proinflammatory cytokine transcription
Source: Nat Commun. 2016 May 23;7:11624. doi: 10.1038/ncomms11624 (PMC4879264; doi:10.1038/ncomms11624)
Supplement: Peer Review File [file ncomms11624-s2.pdf]

Editorial Note: this manuscript has been previously reviewed at another journal that is not operating a transparent peer review scheme. It was accepted without further rounds of review at Nature Communications.
